# Tailoring topological altermagnetic spin texture via interfacial exchange coupling in quasi-2D CrSb/(Bi, Sb)_2_Te_3_ thin film

**DOI:** 10.1038/s41467-026-72021-7

**Published:** 2026-04-17

**Authors:** Peng Chen, Josep Ingla-Aynés, Chen Chang, Eugene Park, Don Heiman, Frances M. Ross, Hang Chi, Jagadeesh S. Moodera

**Affiliations:** 1 Francis Bitter Magnet Laboratory, Plasma Science and Fusion Center, Massachusetts Institute of Technology, Cambridge, MA USA; 2 Department of Materials Science and Engineering, Massachusetts Institute of Technology, Cambridge, MA USA; 3 Department of Physics, Northeastern University, Boston, MA USA; 4 Department of Physics, University of Ottawa, Ottawa, ON Canada; 5 School of Electrical Engineering and Computer Science, University of Ottawa, Ottawa, ON Canada; 6 Nexus for Quantum Technologies, University of Ottawa, Ottawa, ON Canada; 7 Department of Physics, Massachusetts Institute of Technology, Cambridge, MA USA

**Keywords:** Magnetic properties and materials, Topological insulators

## Abstract

Altermagnets featuring collinear magnetic order and momentum-space spin splitting are a newly identified class of magnetically ordered materials. Most studies so far on altermagnetism focus on quasi-3D (e.g., > 10 nm) systems with bulk symmetry-dominance, while the quasi-2D regime with symmetry-breaking remains largely unexplored. Here, we report a pronounced anomalous Hall effect and tunable spin anisotropy arising from symmetry-breaking of the altermagnetic order in CrSb (2.4 nm)/(Bi_1-*x*_Sb_*x*_)_2_Te_3_ heterostructures. The anomalous Hall effect strength, driven by the modulation of altermagnetic spin texture orientation, can be tuned through strain via controlling the thickness and Fermi level of the topological insulator (Bi_1-*x*_Sb_*x*_)_2_Te_3_. The effective Hamiltonian model reveals that the exchange coupling between the altermagnetic order and topology can produce a unique hybrid anomalous Hall effect. Angle-dependent magneto-transport investigation uncovers the magnetic dynamics of altermagnetic moments with exchange coupling. Our strategy broadens the way to explore altermagnetic interfacial physics and topological spintronics in quasi-2D altermagnet/topological insulator heterostructures.

## Abstract (web summary)

The authors display a comprehensive study on the manipulation of topology and altermagnetism by controlling the hybridization of the surface states and the Fermi level of topological insulators.

## Introduction

The integration of magnetism and topologically nontrivial electronic states has opened exciting avenues for realizing novel quantum phases and functionalities in condensed matter systems^1–10^. Magnetic topological insulator (TI) heterostructures provide a promising avenue to engineer topological and uniform magnetic order through the interfacial proximity effect^11–14^. Among the various classes of magnetism, altermagnets (AMs) have recently emerged as a newly recognized class of magnetically ordered materials^15–19^, which exhibit antiferromagnet-like compensated collinear magnetic configurations and ferromagnet-like spin splitting in momentum space. Experimentally, angle-resolved photoemission spectroscopy (ARPES) has directly revealed spin-split electronic structures exhibiting hallmark characteristics of AMs in CrSb^16,20–22^ and MnTe^23,24^. Meanwhile, a non-zero anomalous magneto-transport phenomenon has been observed across various AMs^17,25,26^, further supporting their unique spin-dependent electronic properties. Nevertheless, while prior studies have predominantly addressed altermagnetic behavior in quasi-3D (e.g., larger than 10 nm) or bulk systems with symmetry-dominance, the quasi-2D regime with symmetry-breaking remains relatively unexplored.

In the quasi-2D regime, such as in AM/TI heterostructures, interfacial effects (e.g., strain and exchange coupling) play a central role^14^ and offer unique opportunities to investigate emerging physical phenomena not present in quasi-3D or bulk systems. Correspondingly, AM/TI heterostructures have been theoretically proposed to offer advantages over ferromagnetic or antiferromagnetic TI heterostructures^27,28^. In AM/TI heterostructures, theoretical studies have suggested that the interplay between the spin texture of AMs and the topological surface states (TSSs) of TIs can not only modulate higher-order topological phases within the TI^27^, probing spin polarization of AMs in momentum space^28^, but also provide an opportunity for realizing Majorana modes when integrated with superconductors^29^. Therefore, quasi-2D AM/TI heterostructures serve as a potential platform for the modulation of altermagnetic spin texture and interfacial exchange coupling.

In this article, we report on a pronounced and controllable anomalous Hall effect (AHE) through strain-induced symmetry-breaking and the tunable topological altermagnetic spin texture in quasi-2D CrSb (2.4 nm)/(Bi_1-*x*_Sb_*x*_)_2_Te_3_ thin films grown by molecular beam epitaxy (MBE). The TI-thickness- and Sb-content-dependent magneto-transport results with various (Bi_1-*x*_Sb_*x*_)_2_Te_3_ layers reveal a modified altermagnetic order due to interfacial strain, leading to a two-step AHE response. The observation has been attributed to the coupling interaction between the altermagnetic moment of CrSb and the topological spin texture of the TI, which is supported by an effective Hamiltonian model. Furthermore, the control of Sb-content in (Bi_1-*x*_Sb_*x*_)_2_Te_3_ allows for the modulation of the topological altermagnetic spin texture, as seen in the angle-dependent AHE responses with the tunable two-step hysteresis loop. In addition, the magnetic dynamics of the effective altermagnetic moment contributed from CrSb can be described by the modified Kondorsky model. Our systematic investigation thus highlights the importance of strain and exchange coupling effects in tailoring the topological altermagnetic spin texture. These insights may enable the design of interfacial topological altermagnetic spin texture coupling, facilitate the development of related spin-orbit torque devices, and further integrate altermagnetic superconducting/topological superconducting states in the quasi-2D AM/TI regime.

## Results

**Fig. 1:**
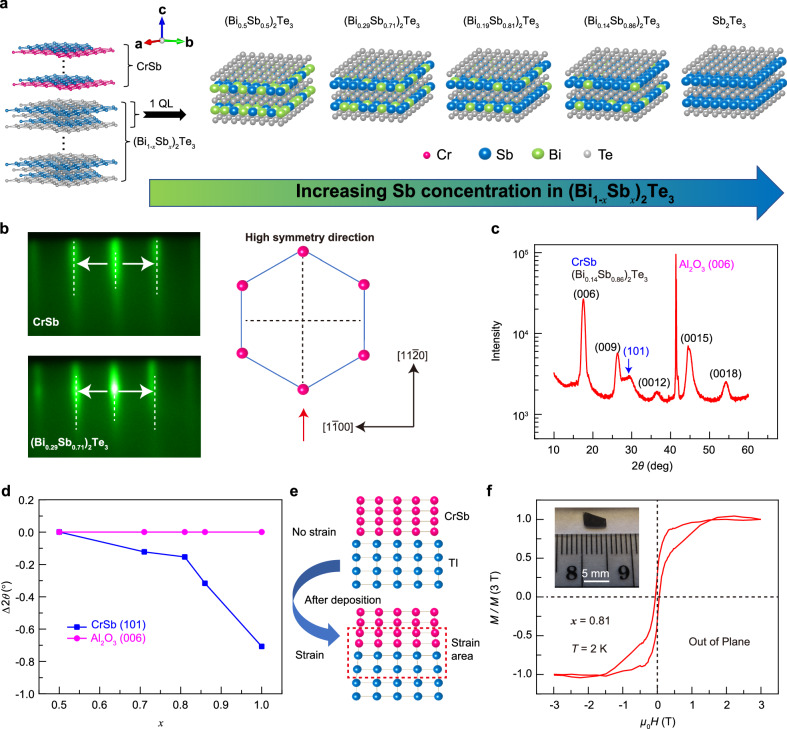
Structural and magnetization characterizations of the MBE-grown CrSb/(Bi_1-*x*_Sb_*x*_)_2_Te_3_ thin films. **a** Illustration of CrSb/(Bi_1-*x*_Sb_*x*_)_2_Te_3_ bilayers with varied Sb-to-Bi ratios. **b** In-situ RHEED patterns of the (Bi_1-*x*_Sb_*x*_)_2_Te_3_ and CrSb. The sharp patterns are sustained during the entire growth. **c** XRD patterns of the 2.4 nm CrSb/7 QL (Bi_0.14_Sb_0.86_)_2_Te_3_ thin film grown on the Al_2_O_3_ (001) substrate. **d** Shift of XRD peak position of Al_2_O_3_ (006) and CrSb (101) as a function of Sb content *x*. Here, Δ2*θ* = 2*θ* (*x*) − 2*θ* (*x* = 0.5) is the difference of the 2*θ* positions of CrSb (101) with respect to the *x* = 0.5 sample. **e** The schematic of strain effect due to the mismatch of in-plane lattice constant between CrSb (4.13 Å) and (Bi_1-*x*_Sb_*x*_)_2_Te_3_ (~ 4.26-4.33 Å). **f** SQUID magnetization hysteresis loop of 2.4 nm CrSb/7 QL (Bi_0.19_Sb_0.81_)_2_Te_3_ thin film at *T* = 2 K.

Epitaxial thin film of bilayer CrSb/(Bi_1-*x*_Sb_*x*_)_2_Te_3_ with AlO_*x*_ protection capping layer was grown on Al_2_O_3_ (001) substrate by MBE (X-ray reflectivity, XRR data presented in Supplementary Section 1 were utilized to obtain the CrSb film thickness). For the comprehensive study, as shown in Fig. 1a, a series of CrSb/(Bi_1-*x*_Sb_*x*_)_2_Te_3_ (*x* = 0.5, 0.71, 0.81, 0.86, and 1) samples were grown with different Sb concentrations. During the entire heterostructure growth process, in-situ surface-sensitive reflection high-energy electron diffraction (RHEED) was employed to monitor the growth dynamics. Figure 1b displays the sharp RHEED patterns obtained along a high symmetry direction (e.g., $[11\bar{2}0]$) after the deposition of both hexagonal (Bi_0.29_Sb_0.71_)_2_Te_3_ ($R\bar{3}m$)^30^ and CrSb (P6_3_/*mmc*)^20^ layers, indicating a two-dimensional epitaxial growth mode. The larger reciprocal lattice unit (*d*-spacing) for the 1st-order RHEED streaks in CrSb than that of (Bi_0.29_Sb_0.71_)_2_Te_3_, indicates that the in-plane lattice constant of CrSb (4.13 Å)^31^ is smaller than that of (Bi_0.29_Sb_0.71_)_2_Te_3_ (~4.3 Å)^32^. Meanwhile, the small lattice mismatch (~4%) also rules out CrSb_2_ phases, an orthorhombic marcasite structure (*Pnnm*)^33^ incompatible with the TI, which exhibits more than 50% lattice mismatch. Cross-sectional aberration-corrected scanning transmission electron microscopy (STEM) images showed the expected CrSb and TI atomic structures above and below a generally planar interface, with energy dispersive X-ray spectroscopy (EDS) analysis confirming the absence of secondary phases (see Supplementary Section 2). Figure 1c exhibits the X-ray diffraction (XRD) data for the as-grown 2.4 nm CrSb/ 7 quintuple layer (QL) (Bi_0.14_Sb_0.86_)_2_Te_3_ sample. Following the Bragg equation $2d\sin \theta=n{{{\uplambda }}}$ and the hexagonal interplanar spacing formula $\frac{1}{{d}^{2}}=\frac{4}{3}\frac{{h}^{2}+{hk}+{k}^{2}}{{a}^{2}}+\frac{{l}^{2}}{{c}^{2}}$, the major peaks are dominated by the (Bi_0.14_Sb_0.86_)_2_Te_3_ phase, and the remaining peaks can be indexed for CrSb^34^ and sapphire (the protection AlO_*x*_ layer is known to be amorphous), consistent with previous report^31,35^ (for a detailed discussion see Supplementary Section 3). Figure 1d shows the shift (Δ2*θ*) of the (101) peak^34^ of CrSb film grown on (Bi_1-*x*_Sb_*x*_)_2_Te_3_ as a function of the Sb content (*x*) compared to the sapphire substrate (006) peak. As one expects, the Al_2_O_3 _(006) peak remains unchanged. In contrast, the CrSb (101) peak position monotonically shifts to lower angles with the increasing Sb content in the TI. This implies that the interfacial strain in CrSb film sensitively depends on the Sb content *x* in (Bi_1-*x*_Sb_*x*_)_2_Te_3_, which serves as the underlying layer for CrSb growth. This tunable lattice parameter and the resulting strain modify the Cr spin texture. As schematically shown in Fig. 1e, in the absence of strain, the magnetic sublattices in ideal CrSb mutually compensate each other (i.e., *m*_A_ = –*m*_B_, for magnetic moment in sublattices A and B). When strain is introduced during film deposition due to the lattice mismatch, in-plane tensile strain in CrSb may lead to an increase (decrease) in the in-plane (out-of-plane) Cr-Sb bond length, resulting in *m*_A_ ≠ –*m*_B_. This lattice distortion-induced strain disrupts the collinear altermagnetic spin symmetry in CrSb at the surface. As one expects, the strain is highest for the bottommost layer, whereas for the subsequent layers of CrSb, it gradually reduces^36^ as schematically shown in Fig. 1e, and we consider the average strain in our analysis. Additionally, the magnetization loop measured using a superconducting quantum interference device (SQUID) of 2.4 nm CrSb/7 QL (Bi_0.19_Sb_0.81_)_2_Te_3_ at *T* = 2 K displays a two-step magnetic feature as shown in Fig. 1f. This suggests the presence of two distinct magnetic phases that undergo magnetization reversal independently. Thus, the overall structural and magnetization characterizations demonstrate a novel two-component behavior in the high crystalline quality MBE-grown CrSb/(Bi_1-*x*_Sb_*x*_)_2_Te_3_ heterostructures, revealing a strain-mediated tunability of its interfacial spin configuration. This is well-suited for exploring the interface-sensitive properties in quasi-2D AM/TI thin films.

**Fig. 2:**
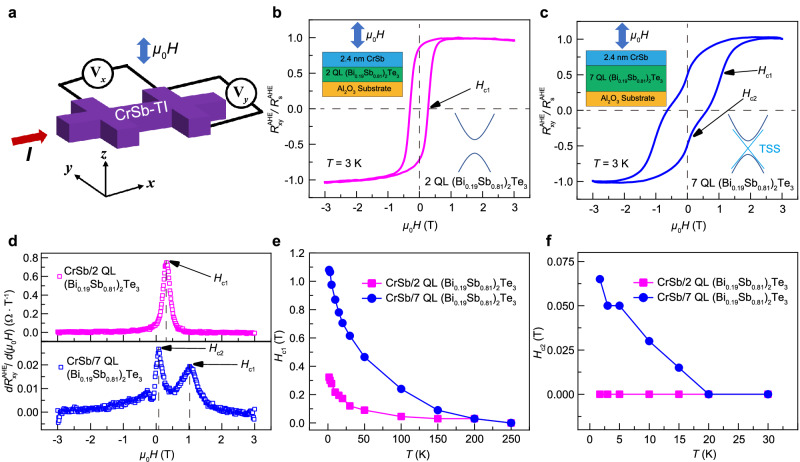
Anomalous Hall response in 2.4 nm CrSb/2 QL (Bi_0.19_Sb_0.81_)_2_Te_3_ and 2.4 nm CrSb/7 QL (Bi_0.19_Sb_0.81_)_2_Te_3_ thin films. **a** Schematic of the six-terminal Hall bar device and the magneto-transport measurement setup. The input current *I* and magnetic field *μ*_0_*H* are applied along the *x*- and *z*-axis, respectively. **b** Normalized anomalous Hall resistance of the 2.4 nm CrSb/2 QL (Bi_0.19_Sb_0.81_)_2_Te_3_ sample at *T* = 3 K. **c** Normalized hybrid anomalous Hall response including two coercivities (*H*_c1_ and *H*_c2_) of the 2.4 nm CrSb/7 QL (Bi_0.19_Sb_0.81_)_2_Te_3_ thin film at *T* = 3 K. **d** Derivative of ${R}_{{xy}}^{{{{\rm{AHE}}}}}$ of **b** and **c** as a function of *μ*_0_*H*. The peaks indicate the corresponding coercivities. **e**, **f** Temperature-dependent *H*_c1_ and *H*_c2_ of 2.4 nm CrSb/2 QL (Bi_0.19_Sb_0.81_)_2_Te_3_ and 2.4 nm CrSb/7 QL (Bi_0.19_Sb_0.81_)_2_Te_3_ thin films.

The implication of the above observation is further evidenced in the magneto-transport measurements using the six-terminal Hall bar geometry on devices of 2.4 nm CrSb/2 QL (Bi_0.19_Sb_0.81_)_2_Te_3_ and 2.4 nm CrSb/7 QL (Bi_0.19_Sb_0.81_)_2_Te_3_ thin films. As shown in Fig. 2a, the longitudinal and transverse voltages (*V*_*x*_ and *V*_*y*_) as a function of applied magnetic field *μ*_0_*H* along the *z*-direction were measured with a current excitation. In the anomalous Hall response with ordinary Hall effect (OHE) contribution subtracted (for procedure and the exclusion of geometry-dependent AHE, see Supplementary Sections 4 and 5), the normalized ${R}_{{xy}}^{{{{\rm{AHE}}}}}/{R}_{{{{\rm{s}}}}}^{{{{\rm{AHE}}}}}$ of 2.4 nm CrSb/2 QL (Bi_0.19_Sb_0.81_)_2_Te_3_ at *T* = 3 K (Fig. 2b) displays the traditional ferromagnet-like magnetic response with a single coercivity *H*_c1_, indicating a single magnetic phase. Here, ${R}_{{xy}}^{{{{\rm{AHE}}}}}$ and ${R}_{{{{\rm{s}}}}}^{{{{\rm{AHE}}}}}$ are the anomalous Hall and saturated anomalous Hall resistance, respectively. Further increasing the TI thickness to 4 QL still maintained the single hysteresis loop with a coercive field *H*_c1_ of ~0.780 T (see Supplementary Section 6). In contrast, upon keeping the same thickness of the CrSb layer and increasing the thickness of the (Bi_0.19_Sb_0.81_)_2_Te_3_ layer to 7 QL, a two-step anomalous Hall response with two distinct coercive fields *H*_c1_ and *H*_c2_ emerges (Fig. 2c), mirroring the two-step magnetization observed in SQUID measurements (Fig. 1f). This two-step behavior can still be observed with TI thickness up to 12 QL (see Supplementary Section 6). This behavior suggests the coexistence of strain-broken altermagnetic order and interfacial exchange coupling, resulting in two distinct magnetic phases. To extract coercive fields *H*_c1_ and *H*_c2_ from the hysteresis loop in Fig. 2b-c, magnetic susceptibility $(d({M}_{m})/d({\mu }_{0}H))$ was plotted as a function of the applied magnetic field, where the peaks in this curve correspond to the coercive fields.

In general, the Hall resistance can be expressed as ${R}_{{xy}}={R}_{{xy}}^{{{{\rm{OHE}}}}}+{R}_{{xy}}^{{{{\rm{AHE}}}}}={R}_{0}\cdot {\mu }_{0}H+{R}_{s}\cdot {M}_{m}$, where the first term is the ordinary carrier-driven contribution, whereas the second term represents the AHE contribution from the spontaneous magnetization; ${R}_{0},{R}_{s}$, and ${M}_{m}$ represent ordinary Hall coefficient, anomalous Hall coefficient, and the uncompensated magnetization arising from the breaking of collinear altermagnetic order, respectively^37^. Following this scenario, the coercive fields can also be extracted from the peak positions in the field derivative of the anomalous Hall resistance $[d({R}_{{xy}}^{{{{\rm{AHE}}}}})/d\left({\mu }_{0}H\right){{{\rm{vs.}}}}\,{\mu }_{0}H]$. As displayed in Fig. 2d, 2.4 nm CrSb/2 QL (Bi_0.19_Sb_0.81_)_2_Te_3_ bilayer exhibits a single peak, corresponding to a single coercive field *H*_c1_ (~ 0.300 T) at 3 K, whereas the 2.4 nm CrSb/7 QL (Bi_0.19_Sb_0.81_)_2_Te_3_ bilayer shows two distinct peaks, indicating two coercive fields *H*_c1_ (~ 1.065 T) and *H*_c2_ (~ 0.050 T) at 3 K, associated with the presence of two magnetic phases. The clear hysteresis loops observed in both of them suggest that the broken-collinear altermagnetic spin texture by the strained structure is responsible for the main transport signal in the CrSb/TI system. One observes that the *H*_c1_ for 2.4 nm CrSb/7 QL (Bi_0.19_Sb_0.81_)_2_Te_3_ is larger than that of 2.4 nm CrSb/2 QL (Bi_0.19_Sb_0.81_)_2_Te_3_ until 250 K (Fig. 2e, with the corresponding longitudinal signal of 2.4 nm CrSb/7 QL (Bi_0.19_Sb_0.81_)_2_Te_3_ displayed in Supplementary Section 7). This may be due to different interfacial strain in 2.4 nm CrSb/7 QL (Bi_0.19_Sb_0.81_)_2_Te_3_ and 2.4 nm CrSb/2 QL (Bi_0.19_Sb_0.81_)_2_Te_3_ (see anomalous Hall resistance vs. temperature in Supplementary Section 6). In addition, the transition temperature (defined by the temperature at which coercivity and hysteresis loops disappear) of 2.4 nm CrSb/2 QL (Bi_0.19_Sb_0.81_)_2_Te_3_ (~ 250 K) is higher than that of 7.2 nm CrSb/2 QL (Bi_0.19_Sb_0.81_)_2_Te_3_ (50-100 K) (Supplementary Section 8), indicating that the interfacial effects tend to dominate as the system approaches the 2D limit. Meanwhile, the second coercivity *H*_c2_ for 2.4 nm CrSb/7 QL (Bi_0.19_Sb_0.81_)_2_Te_3_ vanishes beyond 20 K (Fig. 2f), which is attributed to the exchange coupling between CrSb and TI and the resultant topological spin ordering. With further increasing temperature, the single magnetic phase induced by the interfacial strain effect persists to the critical (transition) temperature of 250 K of CrSb, indicating that the uncompensated magnetic moment, originating from the broken collinear altermagnetic order, can persist up to near room temperature.

To understand the physical origin of the anomalous Hall response in such hybrid structures, we built an effective Hamiltonian in momentum (**k**) space as follows^38,39^:

1 $${H}_{{{{\rm{eff}}}}}={D{{{\bf{k}}}}}^{2}I+H=\,{D{{{\bf{k}}}}}^{2}I+\left(\begin{array}{cc}{{\hslash }}{v}_{F}\left({\sigma }_{x}{k}_{y}-{\sigma }_{y}{k}_{x}\right)+{{{{\bf{M}}}}}_{{{{\bf{ex}}}}}^{{{{\bf{AM}}}}}{{\cdot }}{{{\boldsymbol{\sigma }}}} & tI\\ tI & -{{\hslash }}{v}_{F}\left({\sigma }_{x}{k}_{y}-{\sigma }_{y}{k}_{x}\right)+{{{{\bf{M}}}}}_{{{{\bf{ex}}}}}^{{{{\bf{AM}}}}}{{\cdot }}{{{\boldsymbol{\sigma }}}}\end{array}\right)$$

where the parameters D, ${v}_{F}$, ${{\hslash }}$, and $t$ are the quadratic term, the Fermi velocity, reduced Planck constant, and the interaction term between the top and bottom surfaces of the TI layer, respectively. ${{{{\bf{M}}}}}_{{{{\boldsymbol{ex}}}}}^{{{{\bf{AM}}}}}$, **σ**, and *I* represent exchange field originated from collinear-broken AM CrSb, spin Pauli matrices, and the identity matrix. After diagonalization and processing the determinant (details can be found in Supplementary Section 9), the eigenenergy of *H* can be obtained:

2 $${E}_{H}=\,\pm \sqrt{{\left({M}_{{{{\rm{ex}}}}}^{{{{\rm{AM}}}}}\right)}^{2}+{{{{\bf{k}}}}}^{2}+{t}^{2}\pm 2\sqrt{{\left({\left({M}_{{{{\rm{ex}}}}}^{{{{\rm{AM}}}}}\right)}_{{{{\boldsymbol{x}}}}}{k}_{y}-{\left({M}_{{{{\rm{ex}}}}}^{{{{\rm{AM}}}}}\right)}_{{{{\boldsymbol{y}}}}}{k}_{x}\right)}^{2}+{t}^{2}{\left({M}_{{{{\rm{ex}}}}}^{{{{\rm{AM}}}}}\right)}^{2}}}$$

Note that an exchange field from in-plane magnetization only causes the Dirac cone of the TI to undergo a shift or tilt in *k*-space, rather than opening a magnetic gap that generates AHE^40^. Thus, with an out-of-plane magnetization, we find that the eigenvalues of the Hamiltonian at the Γ point (*k* = 0) are

3 $${E}_{H}=\,\pm ({M}_{{{{\rm{ex}}}}}^{{{{\rm{AM}}}}}\pm t)$$

This yields at the original Dirac point an energy gap $\Delta {E}_{H}=2|{M}_{{{{\rm{ex}}}}}^{{{{\rm{AM}}}}}-t|$. In the 2.4 nm CrSb/2 QL TI heterostructure, the strong hybridization interaction (*t*) between the top and bottom surfaces of the ultrathin TI produces a large energy gap^41^, which results in a greatly reduced influence of the average exchange field induced by the CrSb layer, thereby causing transport to be dominated by the intrinsic magnetization of the CrSb layer, and resulting in a single-step hysteresis loop. As the TI thickness increases to the critical transition thickness of 4 QL, the gap induced by hybridization decreases. Furthermore, in the 2.4 nm CrSb/7, 12 QL TI bilayer, the increased TI thickness effectively decouples the top and bottom surfaces^41^, enabling the CrSb layer to couple predominantly with the top surface, which was supported by ARPES and scanning tunnelling microscopy (STM) results on TIs^41,42^. In this case, the exchange field $({M}_{{{{\rm{ex}}}}}^{{{{\rm{AM}}}}})$ opens a magnetic gap in the top surface state, thereby making the exchange-modified top surface state contribute an additional transport signal. Its significance is evident by the emergence of the two-step hysteresis loop, reflecting the combined effects of strain-broken altermagnetic order and exchange-split surface-state-mediated transport. These findings reveal that quasi-2D altermagnetic order not only gives rise to a pronounced and tunable AHE through strain-induced symmetry-breaking but also can couple with topology via the interface-induced exchange field.

**Fig. 3:**
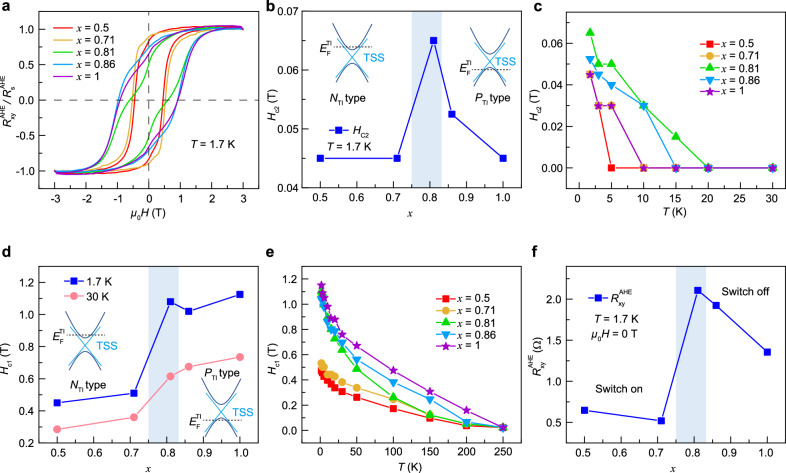
Modulation of the magnetic exchange interaction via engineering the Sb-to-Bi ratio. **a** The normalized ${R}_{{xy}}^{{{{\rm{AHE}}}}}$ displays a hybrid anomalous Hall response in the 2.4 nm CrSb/7 QL (Bi_1-*x*_Sb_*x*_)_2_Te_3_ (*x* = 0.5, 0.71, 0.81, 0.86, and 1) samples at *T* = 1.7 K. **b** The coercivity *H*_c2_ decomposed from **a** exhibits a peak in the case of *x* = 0.81, where the TI contribute *N*_TI_ (*P*_TI_) type electron (hole) carrier when *x* = 0.5 and 0.71 (0.86 and 1). The band schematics illustrate the electron (hole) conduction for *x* < 0.75 (*x* > 0.83). The light blue region indicates that the Fermi level of the TI is located near the Dirac point. **c** Temperature**-**dependent coercivity *H*_c2_ of CrSb/(Bi_1-*x*_Sb_*x*_)_2_Te_3_ (_*x*_ = 0.5, 0.71, 0.81, 0.86, and 1) thin films. **d** The coercivity *H*_c1_ decomposed from **a** and Supplementary Section 11 nearly monotonically enhances upon increasing Sb content. The minor peak of *x* = 0.81 at *T* = 1.7 K may be attributed to the strengthened exchange coupling. **e** Temperature-dependent coercivity *H*_c1_ of CrSb/(Bi_1-*x*_Sb_*x*_)_2_Te_3_ (*x* = 0.5, 0.71, 0.81, 0.86, and 1) samples. **f** Zero-field ${R}_{{xy}}^{{{{\rm{AHE}}}}}$ of CrSb/(Bi_1-*x*_Sb_*x*_)_2_Te_3_ extracted from measured anomalous Hall resistance at *T* = 1.7 K. As Sb increases from 0.71 to 0.81, zero-field ${R}_{{xy}}^{{{{\rm{AHE}}}}}$ increases about 300%.

Given the occurrence of strain-induced AHE and exchange coupling interaction in 2.4 nm CrSb/7 QL (Bi_0.19_Sb_0.81_)_2_Te_3_ thin film, we further investigated the magnetic dynamics of the interfacial exchange-driven spin texture by Fermi level engineering via Sb-doping in (Bi_1-*x*_Sb_*x*_)_2_Te_3_. As shown in Fig. 3a, the normalized ${R}_{{xy}}^{{{{\rm{AHE}}}}}/{R}_{{{{\rm{s}}}}}^{{{{\rm{AHE}}}}}$ of 2.4 nm CrSb/7 QL (Bi_1-*x*_Sb_*x*_)_2_Te_3_ (*x* = 0.5, 0.71, 0.81, 0.86, and 1) thin films at *T* = 1.7 K show a similar two-step anomalous Hall response, while the varied two-step shape is related to the change in exchange coupling strength and the unique interfacial spin texture, consistent with the strained layer seen by the XRD peak shift, indicating strain-broken collinear altermagnetism. To quantitatively visualize the exchange coupling interaction and the strain-induced intrinsic contribution of CrSb, the *H*_c2_ and *H*_c1_ are obtained by the method in Fig. 2d. As illustrated in Fig. 3b, *H*_c2_ for *x* = 0.81 manifests the highest value, suggesting that, when the Fermi level of TI approaches the Dirac point (the Fermi level location in the TI is extracted in Supplementary Section 10), it results in a more pronounced influence, owing to the reduced bulk state contribution. Moreover, for *x* = 0.81, the temperature-dependent *H*_c2_ demonstrates not only the highest value but also has the highest transition temperature (~ 20 K) when the two-step shape occurs, as shown in Fig. 3c. In contrast, with increasing Sb doping, the strain strength for the CrSb (in-plane lattice constant ~4.13 Å^31^) from (Bi_0.5_Sb_0.5_)_2_Te_3_ (~4.33 Å^32^) to Sb_2_Te_3_ (~4.26 Å^32^) shows decreased trend, resulting in increased value of *H*_c1_. As shown in Fig. 3d, the coercivity *H*_c1_ increases by ~150% from (Bi_0.5_Sb_0.5_)_2_Te_3_ to Sb_2_Te_3_. One notices a minor peak at *T* = 1.7 K for *x* = 0.81, and it is absent by *T* = 30 K. While its origin is not understood, it is likely related to the interplay between strain-broken altermagnetic order and the stronger coupling while the Fermi level is close to the Dirac point. Support for this comes from the temperature-dependent *H*_c1_ (see Supplementary Section 11), showing the vanishing of the small peak (Fig. 3e), which coincides with the disappearance of exchange coupling ~ 20 K. As the temperature increases beyond 20 K, *H*_c1_ gradually reverts to a monotonic increase. Furthermore, the zero-field anomalous Hall resistance increases by ~300% as the Sb concentration *x* rises from 0.71 to 0.81, shown in Fig. 3f. This dependence gives control over the topological surface states and thus could allow for a potential transistor-like device as a future direction to be explored. (e.g., by tuning dielectric gating in CrSb/TI heterojunctions). In addition, repeating magneto-transport measurements gave consistent results (*H*_c1_, *H*_c2_, and zero-field anomalous Hall resistance) with standard deviation shown in Supplementary Section 12. Therefore, our results further demonstrate that the tunable altermagnetic order can be induced by strain-modulated symmetry breaking and magnetic exchange coupling in quasi-2D CrSb/(Bi_1-*x*_Sb_*x*_)_2_Te_3_ heterostructure via controllable Fermi level of TI. These observations attest to a promising platform for the development of quasi-2D topological altermagnetic devices.

**Fig. 4:**
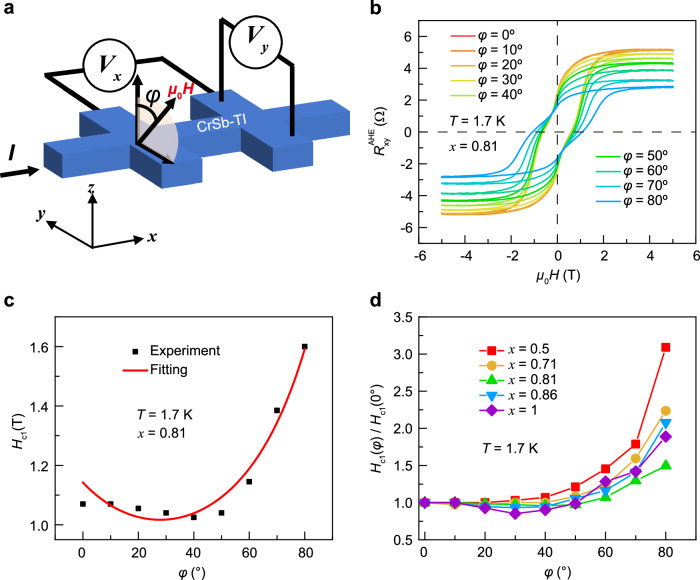
Angle-dependent anomalous Hall response and modified Kondorsky model simulation in CrSb/(Bi_1-*x*_Sb_*x*_)_2_Te_3_ thin films. **a** Schematic of the six-terminal Hall bar device and the magneto-transport measurement setup. The rotation angle *φ* is defined as the angle between the magnetic field direction and *z*-direction. **b** Angle-dependent anomalous Hall resistance of 2.4 nm CrSb/ 7 QL (Bi_0.19_Sb_0.81_)_2_Te_3_ at *T* = 1.7 K. **c** The black squares show the angle-dependent coercivity *H*_c1_ extracted from **b**, and the red line shows the simulated curve based on the modified Kondorsky model. **d** Angle-dependent coercivity *H*_c1_ of 2.4 nm CrSb/ 7 QL (Bi_1-*x*_Sb_*x*_)_2_Te_3_ (*x* = 0.5, 0.71, 0.81, 0.86, and 1) extracted from the measured anomalous Hall transport data.

Furthermore, additional insight into the interplay between magnetic anisotropy and topological surface states in CrSb/(Bi_1-*x*_Sb_*x*_)_2_Te_3_ can be obtained from the angle-dependent magneto-transport properties. Figure 4a shows the experimental setup of the angle-dependent Hall measurement, where the transverse signal (*V*_*y*_) was measured at different angles (*φ* is defined as the angle between the applied magnetic field and the *z*-direction). As shown in Fig. 4b, the anomalous Hall resistance at *T* = 1.7 K, extracted by subtracting the ordinary Hall contribution from the measured Hall signal for the 2.4 nm CrSb/7 QL (Bi_0.19_Sb_0.81_)_2_Te_3_ heterostructure, maintains the pronounced two-step feature at the measured angles from 0° to 80°. This behavior shows that the applied field direction does not significantly perturb the interfacial magnetic exchange coupling. For the study of angular dependence of magnetic dynamics, the Stoner-Wohlfarth (S-W) model^43–45^ and the Kondorsky-type (K-type) model^46–48^ are commonly employed. The S-W model is typically applicable to single-domain magnetic systems^46^, whereas the K-type model is more suitable for systems exhibiting multiple magnetic states or domain structures^46^. In our heterostructure system, the K-type model is utilized to investigate the magnetic dynamic evolution involving distinct magnetic states with different angles as described below^48^:

4 $${H}_{{{{\rm{c}}}}}=\frac{{H}_{{{{\rm{c}}}}}(0^\circ )({N}_{{{{\rm{A}}}}}+{N}_{x})\cos (\varphi )}{{{N}_{z}\sin (\varphi )}^{2}+({N}_{{{{\rm{A}}}}}+{N}_{x}){\cos (\varphi )}^{2}}$$

where the parameters ${H}_{{{{\rm{c}}}}},{N}_{i}(i=x,y,z)$, and ${N}_{{{{\rm{A}}}}}$ represent coercivity, the demagnetizing factor, and magneto-crystalline anisotropy $\left(\right.{N}_{{{{\rm{A}}}}}=\frac{{H}_{{{{\rm{A}}}}}}{{M}_{{{{\rm{S}}}}}},{H}_{{{{\rm{A}}}}}$ and ${M}_{{{{\rm{S}}}}}$ represent anisotropy field and saturation magnetization), respectively. Given the presence of strain and exchange coupling effects in our CrSb/TI heterostructure, the K-type model can be modified as below (see Supplementary Section 13 for detailed discussion):

5 $${H}_{{{{\rm{c}}}}1}=\frac{{H}_{{{{\rm{c}}}}1}(0^\circ ){N}_{{{{\rm{A}}}}}\cos (\varphi+{\varphi }_{0})}{{\sin (\varphi+{\varphi }_{0})}^{2}+{N}_{{{{\rm{A}}}}}{\cos (\varphi+{\varphi }_{0})}^{2}}$$

where the effective angle ${\varphi }_{0}$ accounts for the tilt in the magnetization direction of Cr spin moment away from the *z*-axis under a combination of strain, interface coupling, and other effects. As shown in Fig. 4c, the experimental coercivities ${H}_{{{{\rm{c}}}}1}$ of CrSb for 2.4 nm CrSb/7 QL (Bi_0.19_Sb_0.81_)_2_Te_3_ bilayer at different angles extracted from Fig. 4b can be fitted well at *T* = 1.7 K based on the modified K-type model (see Supplementary Section 14 for fitting at different temperatures). The overall trend is that the tilt angle ${\varphi }_{0}$ gradually diminishes with increasing temperature (see Supplementary Section 14), which supports that strain and exchange coupling effects disrupt the collinear altermagnetic order, leading to a tilting tendency in the spin texture of altermagnetism. As a comparison, the classical K-model and S-W model fitting results and the residuals analysis are provided in Supplementary Section 15. In addition, ${H}_{{{{\rm{c}}}}1}(\varphi )$ exhibits minimum change from *φ* = 0° to 80° at *x* = 0.81, as shown in Fig. 4d. This implies the emergence of stronger interfacial exchange coupling when the Fermi level of the TI approaches the Dirac point (see Supplementary Section 16 for further discussions), better stabilizing altermagnetic order with symmetry breaking.

## Discussion

In conclusion, our results on quasi-2D CrSb demonstrate that pronounced AHE arises from strain-induced modification of collinear altermagnetic spin symmetry. The unique two-step AHE originates from the interfacial exchange coupling between the altermagnetic order of CrSb and the spin-momentum-locked surface spin texture of the TI. The AHE strength arising from quasi-2D altermagnetic order and interfacial topological altermagnetic spin texture can be effectively tailored via engineered surface state hybridization and Fermi level modulation in (Bi_1-*x*_Sb_*x*_)_2_Te_3_. The observed angle-dependent AHE response is consistent with the modified Kondorsky-type model. With further exploration of altermagnetism via modified symmetry, our result provides critical insight into quasi-2D altermagnetism with broken collinear symmetry to achieve the integration of altermagnetic order and topology. This offers a potential route for manipulating magnetic phase transitions and spin textures in altermagnetic topological heterostructures via optimizing interface engineering, and could allow future exploration of intrinsic altermagnetic topological insulators and altermagnetic topological superconductors.

## Methods

### Samples Growth and Characterizations

The CrSb/(Bi_1-*x*_Sb_*x*_)_2_Te_3_ samples (*x* = 0.5, 0.71, 0.81, 0.86, and 1) were synthesized on Al_2_O_3_ (001) substrates using MBE under an ultra-high vacuum of 10^-9 ^Torr. Prior to growth, the Al_2_O_3_ substrates were pre-annealed at 800 °C to ensure surface cleanliness and quality. During deposition, high-purity Bi (99.999%), Sb (99.9999%), and Te (99.999%) were co-evaporated from standard Knudsen cells, and Cr (99.999%) was evaporated from an electron-beam source. The Bi-to-Sb ratio was controlled and verified using a quartz crystal monitor. The (Bi_1-*x*_Sb_*x*_)_2_Te_3_ and CrSb layers were grown sequentially at substrate temperatures of 230 °C and 250 °C, respectively. The growth was followed by a moderate post-growth annealing process at 250 °C to improve the quality. Throughout growth, in-situ RHEED was employed to monitor surface crystallinity and growth dynamics. Subsequently, XRD analysis was conducted to evaluate the crystal structure of the synthesized samples.

### Cross-Sectional Scanning Transmission Electron Microscopy Characterization

Cross-sectional STEM specimens were prepared using a FEI Helios Nanolab 660 DualBeam focused ion beam tool. Carbon and platinum were deposited as protection layers over the sample to minimize ion-beam damage during focused Ga^+^ ion beam milling. A lamella was milled from the region of interest at 30 kV Ga⁺, lifted out using an Omniprobe nanomanipulator, and mounted on a copper TEM half grid (Ted Pella). Final thinning was performed at 2 kV Ga⁺ to minimize Ga implantation and surface amorphization.

High-resolution imaging was carried out using an aberration-corrected Thermo Fischer Themis Z G3 STEM operated at 300 kV with a convergence angle of 25 mrad and a beam current of 55 pA. Low magnification images were acquired using an image size of 2048×2048 pixels with 2 μs/pixel dwell time. High magnification images were acquired using an image size of 1024×1024 pixels with a dwell time of 500 ns/pixel. A series of 10 images was acquired and drift-corrected using the drift correction frame integration (DCFI) function in the microscope’s Velox software to increase the signal-to-noise ratio. EDS was conducted on the same instrument at 300 kV. Elemental maps (atomic %) were acquired with an image size of 1024 × 1024 pixels, a dwell time of 4 μs/pixel, and 65-frame stacking. A Gaussian blur (σ = 1.3) was applied for visualization to enhance map contrast.

### Transport Measurements

Magneto-transport measurements of the CrSb/(Bi_1-*x*_Sb_*x*_)_2_Te_3_-based devices were carried out using a Quantum Design Physical Property Measurement System (PPMS), which enables temperature control down to 1.7 K and magnetic fields up to ±9 T. During the measurements, a typical AC current of 10 μA was applied to the fabricated Hall bar devices. The longitudinal (*V*_*x*_) and transverse (*V*_*y*_) voltages were recorded using a standard lock-in detection technique. To exclude the heating effect, we also carried out magneto-transport measurements on the heterostructure under different currents (in the range of 1–100 μA), with corresponding results in Supplementary Section 17.

## Supplementary information

Supplementary information

Transparent Peer Review file

## Source data

Source Data

## Data Availability

Data generated in this study are provided in the Source Data file or upon request from the corresponding authors. Source data are provided with this paper.
