# Supplementary material for: Tailoring topological altermagnetic spin texture via interfacial exchange coupling in quasi-2D CrSb/(Bi, Sb)2Te3 thin film
Source: Nat Commun. 2026 Apr 17;17:5350. doi: 10.1038/s41467-026-72021-7 (PMC13272750; doi:10.1038/s41467-026-72021-7)
Supplement: Supplementary file 1 — Supplementary information [file 41467_2026_72021_MOESM1_ESM.pdf]

Supplementary Information for Tailoring topological altermagnetic spin texture via interfacial exchange coupling in quasi-2D CrSb/(Bi, Sb)$_2$Te$_3$ thin film

Peng Chen[1]*, Josep Ingla-Aynés[1], Chen Chang[2], Eugene Park[2], Don Heiman[1,3], Frances M. Ross[2], Hang Chi[4,5,6]*, & Jagadeesh S. Moodera[1,7]*

[1]*Francis Bitter Magnet Laboratory, Plasma Science and Fusion Center, Massachusetts Institute of Technology, Cambridge, Massachusetts 02139, USA*

[2]*Department of Materials Science and Engineering, Massachusetts Institute of Technology, Cambridge, Massachusetts 02139, USA*

[3]*Department of Physics, Northeastern University, Boston, Massachusetts 02115, USA*

[4]*Department of Physics, University of Ottawa, Ottawa, Ontario K1N 6N5, Canada*

[5]*School of Electrical Engineering and Computer Science, University of Ottawa, Ottawa, Ontario K1N 6N5, Canada*

[6]*Nexus for Quantum Technologies, University of Ottawa, Ottawa, Ontario K1N 6N5, Canada*

[7]*Department of Physics, Massachusetts Institute of Technology, Cambridge, Massachusetts 02139, USA*

Section 1. CrSb thickness verification via XRR.

Section 2. Cross-sectional electron microscopy characterization of the MBE-grown CrSb/TI thin films.

Section 3. XRD peak calculations of the MBE-grown 2.4 nm CrSb/7 QL (Bi$_{0.19}$Sb$_{0.81}$)$_2$Te$_3$ sample.

Section 4. The ordinary Hall extraction.

Section 5. Confirming geometry-independent AHE.

Section 6. Temperature-dependent anomalous Hall resistance in 2.4 nm CrSb/(Bi$_{0.19}$Sb$_{0.81}$)$_2$Te$_3$ heterostructure with different TI thickness (2, 4, 7, 12 QL).

Section 7. Temperature-dependent longitudinal signals of 2.4 nm CrSb/7 QL (Bi$_{0.19}$Sb$_{0.81}$)$_2$Te$_3$

Section 8. Temperature-dependent anomalous Hall resistance in the 7.2 nm CrSb/2 QL (Bi$_{0.19}$Sb$_{0.81}$)$_2$Te$_3$ heterostructure.

Section 9. The effective Hamiltonian model of CrSb/TI heterostructure.

Section 10. Confirmation of the Fermi level of topological insulator (Bi$_{1-x}$Sb$_x$)$_2$Te$_3$.

Section 11. Hybrid anomalous Hall resistance in the CrSb/(Bi$_{1-x}$Sb$_x$)$_2$Te$_3$ heterostructure with

different TI carrier types.

Section 12. Reproducibility and statistics for $CrSb/(Bi_{1-x}Sb_x)_2Te_3$

Section 13. The modified Kondorsky-type model.

Section 14. Modified K-model fitting under variable temperatures.

Section 15. Comparison of the classical K-model, modified K-model, and S-W model.

Section 16. Derivation of the effective Stoner-Wohlfarth model.

Section 17. Ruling out the current-induced heating effect.

Section 1. CrSb thickness verification via XRR.

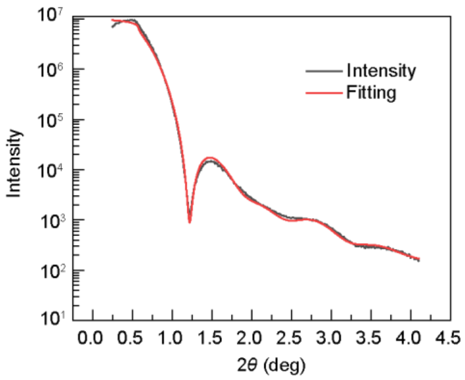

Figure S1. X-ray reflectivity measurement of 8.6 nm CrSb/2 QL TI.

To obtain the growth rate of CrSb, we grew thicker CrSb on 2 QL TI. Subsequently, we carried out X-ray reflectivity (XRR) measurement on the sample. As shown in Fig. S1, by fitting the experimental data, we determined the thickness of the CrSb film to be around 8.6 nm. Considering the total growth time of CrSb (~ 90 minutes), we can estimate the growth rate per monolayer, which in turn enables precise control over the growth of quasi-2D CrSb.

Section 2. Cross-sectional electron microscopy characterization of the MBE-grown CrSb/TI thin films.

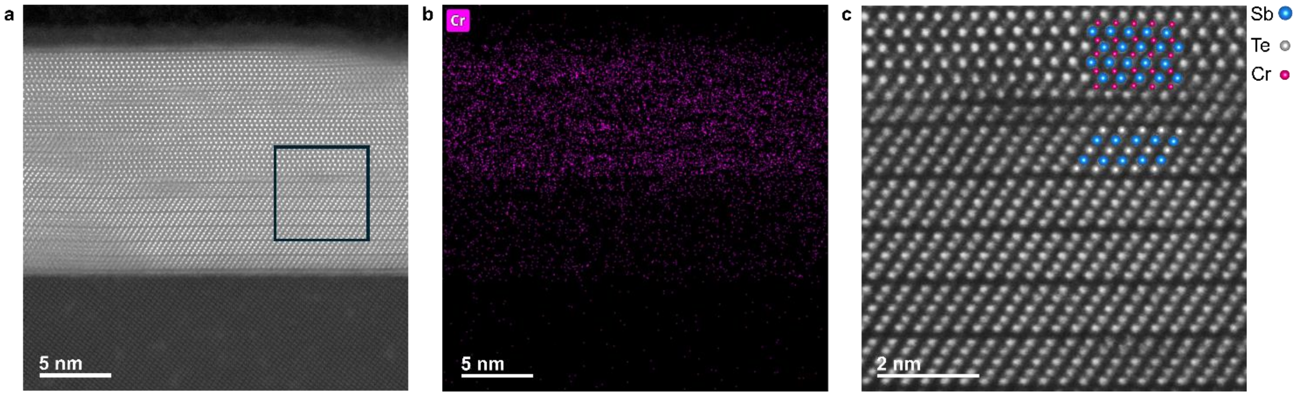

Figure S2. STEM characterization of CrSb/Sb_2Te_3 thin films. a, Cross-sectional high angle annular dark field STEM image of a CrSb/Sb_2Te_3 heterostructure. **b**, the corresponding energy dispersive X-ray analysis of the Cr distribution in the film, showing uniform Cr concentration in the upper layer. **c**, Enlargement of the box indicated in **a** with contrast increased to show details of the atomic arrangement at the interface.

To investigate the crystal structure and defects within our MBE-grown CrSb/TI, we performed aberration-corrected scanning transmission electron microscopy of cross sections of the films prepared using focused ion beam milling. Figure S2a shows the atomic arrangements above and below the CrSb/TI interface. The interface is planar, although growth defects are present elsewhere in the CrSb layer. Figure S2c shows the interface structure in more detail, including an interfacial misfit dislocation. The uniformity of the Cr distribution is shown in Fig. S2b.

Section 3. XRD peak calculations of the MBE-grown 2.4 nm CrSb/7 QL $(Bi_{0.19}Sb_{0.81})_2Te_3$ sample.

To obtain the crystalline phase from XRD, we can adopt the Bragg equation[1] $2d\sin\theta = n\lambda$, where d is the interplanar spacing, λ = 1.5406 Å is the incident X-ray wavelength, θ is the angle between the incident X-ray and the corresponding crystal plane, and n is the diffraction order, respectively. For the hexagonal structure, the interplanar spacing theoretical calculation formula[2] can be adopted by $\frac{1}{d^2} = \frac{4}{3}\frac{h^2+hk+k^2}{a^2} + \frac{l^2}{c^2}$, where the h, k, and l are Miller indices, a and c are in-plane and out-of-plane lattice constants, respectively. Combining the experimental results and theoretical calculations, the crystalline phase corresponding to the peak of XRD can be confirmed. For example, based on the peak position of CrSb (101) in Fig. 1c of the main text, n can be chosen as 1. Therefore, the interplanar spacing can be obtained by the Bragg equation as d ~ 3.083 Å, in agreement with the theoretical interplanar spacing d ~ 3.002 Å obtained using a ~ 4.13 Å and c ~ 5.52 Å[3,4]. According to this scenario, we can get the thin film crystal phase corresponding to the XRD peak in Fig. 1c of the main text.

Section 4. The ordinary Hall extraction.

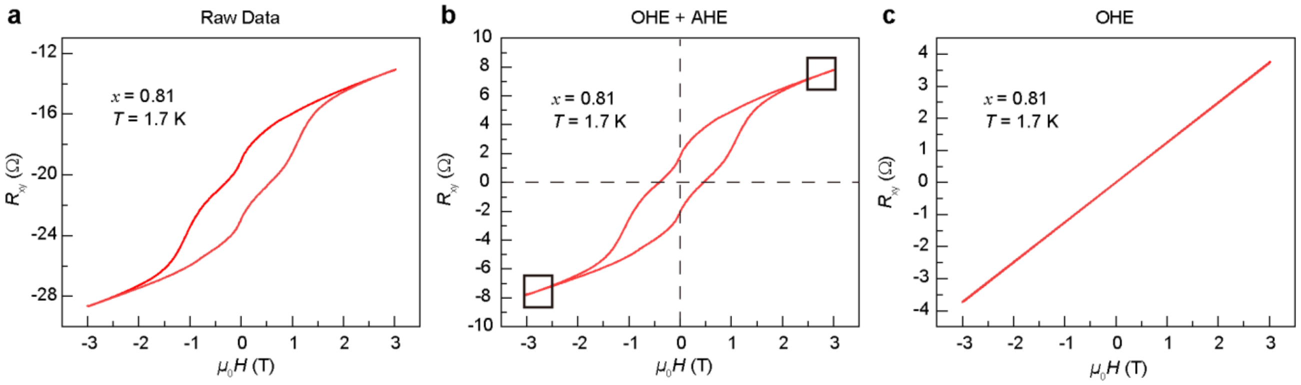

Figure S3. Extracted the ordinary Hall contribution of 2.4 nm CrSb/7QL $(Bi_{0.19}Sb_{0.81})_2Te_3$. a, The raw data of 2.4 nm CrSb/7QL $(Bi_{0.19}Sb_{0.81})_2Te_3$. **b**, the Ordinary Hall effect (OHE) and AHE contribution after subtracting the longitudinal contribution. **c**, the OHE contribution via high magnetic field fitting marked in two black boxes of **b**.

We carried out Hall transport experiments. A portion of longitudinal signals often gets picked up during transverse measurements due to electrode asymmetry caused by fabrication. The usual procedure is to antisymmetrize $R_{xy} = (R_{xy}^{Raw}(+\mu_0H)-R_{xy}^{Raw}(-\mu_0H))/2$ to eliminate the longitudinal signal[5], as shown in Figs. S3a-b. To extract the ordinary Hall component from R_{xy}, the ordinary Hall effect (OHE) coefficient R_0 is obtained by fitting the slope of R_{xy} at high magnetic fields (two black boxes in Fig. S3b). Subsequently, the OHE component $R_0*\mu_0H$ can be further obtained, as shown in Fig. S3c. Accordingly, the AHE contribution $R_{xy}^{AHE} = R_{xy} - R_0*\mu_0H$ can be captured.

Section 5. Confirming geometry-independent AHE.

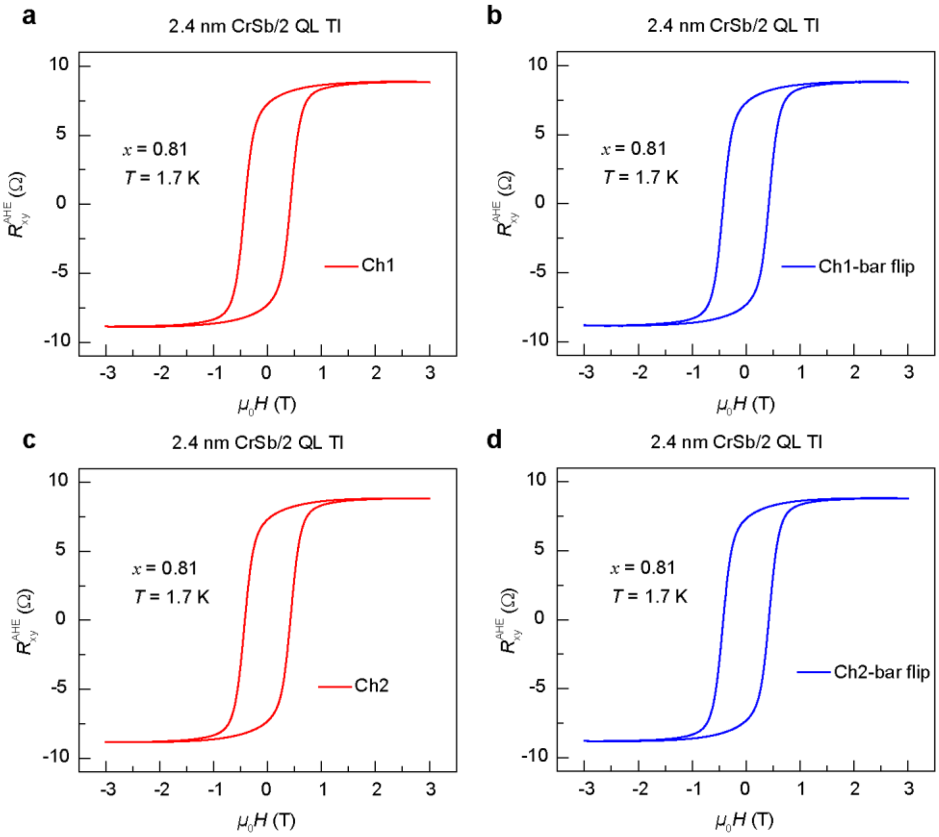

Figure S4. Magnetic field-dependent AHE based on channels 1, 1-bar flip (180° rotated), 2, and 2-bar flip (180° rotated) of 2.4 nm CrSb/2 QL $(Bi_{0.19}Sb_{0.81})_2Te_3$.

We carried out magneto-transport measurements on channels 1, 1-bar flip (180° rotated), 2, and 2-bar flip (180° rotated) of 2.4 nm CrSb/2 QL $(Bi_{0.19}Sb_{0.81})_2Te_3$. As shown in Fig. S4, all of the AHE signals display similar hysteresis loops, which can confirm geometry-independent AHE.

Section 6. Temperature-dependent anomalous Hall resistance in 2.4 nm $CrSb/(Bi_{0.19}Sb_{0.81})_2Te_3$ heterostructure with different TI thickness (2, 4, 7, 12 QL).

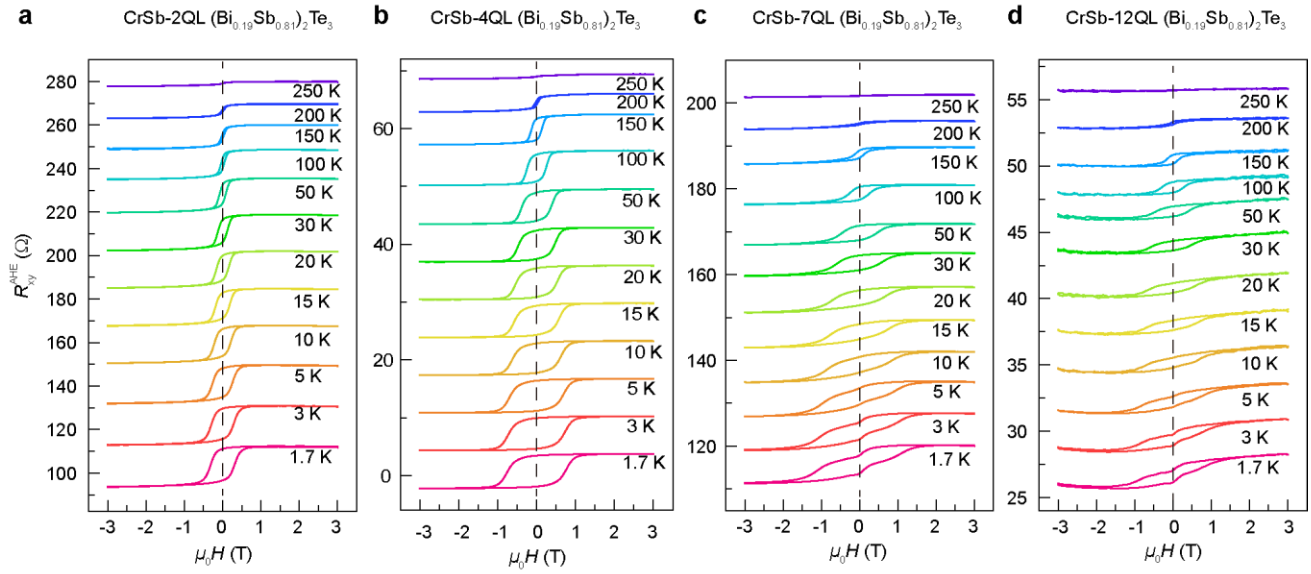

Figure S5. Temperature-dependent anomalous Hall resistance. a-d, Temperature dependence of anomalous Hall resistance of 2.4 nm $CrSb/(Bi_{0.19}Sb_{0.81})_2Te_3$ with various TI thicknesses of 2, 4, 7, 12 QL from 1.7 K to 250 K.

Figure S5 presents the anomalous Hall hysteresis loops of 2.4 nm $CrSb/(Bi_{0.19}Sb_{0.81})_2Te_3$ with different TI thickness (2, 4, 7, 12 QL) across a temperature range from 1.7 K to 250 K. Fig. S5a exhibits a conventional ferromagnetic-like hysteresis loop for the 2 QL TI characterized by a single magnetic phase, with the coercive field progressively decreasing as temperature increases, eventually leading to the complete disappearance of the hysteresis loop at 250 K. As TI thickness increases from 2 QL to 4 QL (Fig. S5b), the single coercivity gradually increases to 0.78 T at 1.7 K, at which point no obvious two-step behavior is observed. In contrast, with TI thickness further increases to 7 QL and 12 QL, Figs. S5c-d display a distinct two-step anomalous Hall hysteresis at low temperatures (below 20 K), indicating the presence of multiple magnetic phases. As the temperature increases, this two-step feature gradually vanishes into a single-phase ferromagnetic-like hysteresis loop above 20 K, which ultimately disappears at 250 K.

Section 7. Temperature-dependent longitudinal signals of 2.4 nm CrSb/7 QL $(Bi_{0.19}Sb_{0.81})_2Te_3$

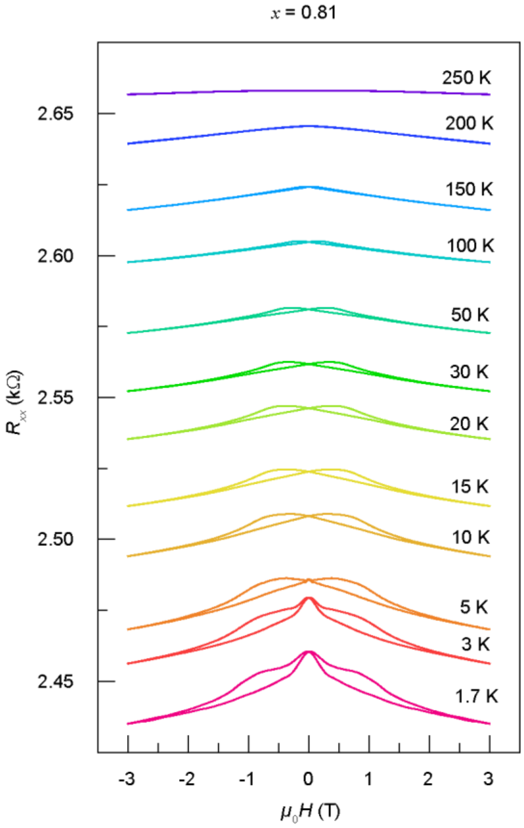

Figure S6. The longitudinal signals of 2.4 nm CrSb/7 QL $(Bi_{0.19}Sb_{0.81})_2Te_3$ at different temperatures.

Figure S6 displays the longitudinal resistance signal (R_{xx}) of 2.4 nm CrSb/7 QL $(Bi_{0.19}Sb_{0.81})_2Te_3$, wherein the hybrid feature of R_{xx} vanishes at a temperature approximately between 10 K and 15 K. Although this temperature deviates slightly from the temperature at which H_{c2} vanishes in R_{xy}, a transition from a composite R_{xx} to a single R_{xx} is observed. As the temperature rises further, the butterfly-type signal in single R_{xx} disappears at approximately 250 K, which is consistent with the temperature at which H_{c1} vanishes.

Section 8. Temperature-dependent anomalous Hall resistance in the 7.2 nm CrSb / 2 QL $(Bi_{0.19}Sb_{0.81})_2Te_3$ heterostructure.

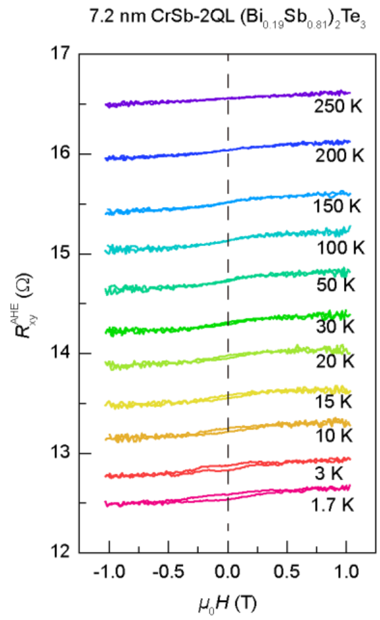

Figure S7. Temperature-dependent anomalous Hall resistance in the 7.2 nm CrSb/2 QL $(Bi_{0.19}Sb_{0.81})_2Te_3$

To confirm the quasi-2D CrSb-dominated interfacial effects in our transport studies, we carried out magneto-transport experiments on 7.2 nm CrSb/2 QL $(Bi_{0.19}Sb_{0.81})_2Te_3$. We observe that with thicker CrSb, the interfacial effect decreases, due to a greater contribution of CrSb with reduced strain. As shown in Fig. S7, the temperature-dependent anomalous Hall behavior shows a reduced transition temperature (50-100 K), indicating that the CrSb with reduced strain dominates the contribution of interfacial effects.

Section 9. The effective Hamiltonian model of CrSb/TI heterostructure.

To better understand the physical origin of the hybrid anomalous Hall response, we can build the effective Hamiltonian in moment space ($\mathbf{k}$ space) as follows[6,7]:

$$H_{\text{eff}} = D\mathbf{k}^2 I + H = D\mathbf{k}^2 I + \begin{pmatrix} \hbar v_F(\sigma_x k_y - \sigma_y k_x) + \mathbf{M}_{ex}^{\mathbf{AM}} \cdot \boldsymbol{\sigma} & tI \\ tI & -\hbar v_F(\sigma_x k_y - \sigma_y k_x) + \mathbf{M}_{ex}^{\mathbf{AM}} \cdot \boldsymbol{\sigma} \end{pmatrix} \quad (1)$$

The parameters D, v_F, $\hbar$, and t are the quadratic term, the Fermi velocity, reduced Planck constant, and interaction term between the top and bottom surfaces of the TI layer, respectively. $\mathbf{M}_{ex}^{\mathbf{AM}}$, $\boldsymbol{\sigma}$, and I represent exchange field originated from altermagnet CrSb with noncollinearity, spin matrices, and the identity matrix, respectively. To diagonalize and simplify the model, the quadratic term can be neglected. Meanwhile, we can set $h_R = \sigma_x k_y - \sigma_y k_x$, $\hbar v_F = 1$. So, the model can be rewritten as

$$H = \begin{pmatrix} h_R + \mathbf{M}_{\mathbf{ex}}^{\mathbf{AM}} \cdot \boldsymbol{\sigma} & tI \\ tI & -h_R + \mathbf{M}_{\mathbf{ex}}^{\mathbf{AM}} \cdot \boldsymbol{\sigma} \end{pmatrix} \quad (2)$$

The Pauli matrices $\boldsymbol{\sigma}$ in the Hamiltonian model are not easy to diagonalize directly, so we consider H^2.

$$H^2 = \begin{pmatrix} A+B & tI \\ tI & A-B \end{pmatrix}^2 = \begin{pmatrix} A^2 + B^2 + \{A,B\} + t^2 I & 2tAI \\ 2tAI & A^2 + B^2 - \{A,B\} + t^2 I \end{pmatrix} \quad (3)$$

where $A = \mathbf{M}_{\mathbf{ex}}^{\mathbf{AM}} \cdot \boldsymbol{\sigma} = (M_{\text{ex}}^{\text{AM}})_x \sigma_x + (M_{\text{ex}}^{\text{AM}})_y \sigma_y + (M_{\text{ex}}^{\text{AM}})_z \sigma_z$ and $B = h_R = \sigma_x k_y - \sigma_y k_x$. So, $A^2 = \left((M_{\text{ex}}^{\text{AM}})_x^2 + (M_{\text{ex}}^{\text{AM}})_y^2 + (M_{\text{ex}}^{\text{AM}})_z^2\right) I = (M_{\text{ex}}^{\text{AM}})^2 I$, $B^2 = (k_x^2 + k_y^2) I = k^2 I$, $\{A, B\} = \{(M_{\text{ex}}^{\text{AM}})_x \sigma_x + (M_{\text{ex}}^{\text{AM}})_y \sigma_y + (M_{\text{ex}}^{\text{AM}})_z \sigma_z, \sigma_x k_y - \sigma_y k_x\} = 2((M_{\text{ex}}^{\text{AM}})_x k_y - (M_{\text{ex}}^{\text{AM}})_y k_x) I$. Then, we obtain:

$$H^2 =$$

$$\begin{pmatrix} ((M_{\text{ex}}^{\text{AM}})^2 + k^2 + t^2) I + 2\left((M_{\text{ex}}^{\text{AM}})_x k_y - (M_{\text{ex}}^{\text{AM}})_y k_x\right) I & 2t\mathbf{M}_{\mathbf{ex}}^{\mathbf{AM}} \cdot \boldsymbol{\sigma} \\ 2t\mathbf{M}_{\mathbf{ex}}^{\mathbf{AM}} \cdot \boldsymbol{\sigma} & ((M_{\text{ex}}^{\text{AM}})^2 + k^2 + t^2) I - 2\left((M_{\text{ex}}^{\text{AM}})_x k_y - (M_{\text{ex}}^{\text{AM}})_y k_x\right) I \end{pmatrix}$$

(4)

Assuming the eigenvalue of H^2 is λ_H^2, we can find $\det(H^2 - \lambda_H^2 I) = 0$. Through the block matrix determinant formula, we can get:

$$\begin{vmatrix} \left((M_{\mathrm{ex}}^{\mathrm{AM}})^2 + k^2 + t^2 - \lambda_H^2\right) + 2\left((M_{\mathrm{ex}}^{\mathrm{AM}})_x k_y - (M_{\mathrm{ex}}^{\mathrm{AM}})_y k_x\right) & 2t(M_{\mathrm{ex}}^{\mathrm{AM}}) \cdot \sigma \\ 2t(M_{\mathrm{ex}}^{\mathrm{AM}}) \cdot \sigma & \left((M_{\mathrm{ex}}^{\mathrm{AM}})^2 + k^2 + t^2 - \lambda_H^2\right) - 2\left((M_{\mathrm{ex}}^{\mathrm{AM}})_x k_y - (M_{\mathrm{ex}}^{\mathrm{AM}})_v k_x\right) \end{vmatrix}$$

$$= \left((M_{\mathrm{ex}}^{\mathrm{AM}})^2 + k^2 + t^2 - \lambda_H^2\right)^2 - 4\left((M_{\mathrm{ex}}^{\mathrm{AM}})_x k_y - (M_{\mathrm{ex}}^{\mathrm{AM}})_y k_x\right)^2 - 4t^2 (M_{\mathrm{ex}}^{\mathrm{AM}})^2 = 0 \qquad (5)$$

Therefore, the eigenvalue of H^2 is

$\lambda_H^2 = (M_{\mathrm{ex}}^{\mathrm{AM}})^2 + k^2 + t^2 \pm 2\sqrt{\left((M_{\mathrm{ex}}^{\mathrm{AM}})_x k_y - (M_{\mathrm{ex}}^{\mathrm{AM}})_y k_x\right)^2 + t^2 (M_{\mathrm{ex}}^{\mathrm{AM}})^2}$. By simplification, the eigenenergy of H can be obtained as:

$$E_H = \pm\lambda_H = \pm\sqrt{(M_{\mathrm{ex}}^{\mathrm{AM}})^2 + k^2 + t^2 \pm 2\sqrt{\left((M_{\mathrm{ex}}^{\mathrm{AM}})_x k_y - (M_{\mathrm{ex}}^{\mathrm{AM}})_y k_x\right)^2 + t^2 (M_{\mathrm{ex}}^{\mathrm{AM}})^2}} \qquad (6)$$

Considering an in-plane magnetization to the surface states of a TI, it causes the Dirac cone of the TI to undergo a shift or tilt in *k*-space, rather than opening a magnetic gap that generates AHE[8]. Thus, with an out-of-plane magnetization, we find that the eigenvalues of the Hamiltonian at the Γ point (k = 0) are

$$E_H = \pm((M_{\mathrm{ex}}^{\mathrm{AM}}) \pm t) \qquad (7)$$

at the original Dirac point, providing an energy gap $\Delta E_H = 2|(M_{\mathrm{ex}}^{\mathrm{AM}}) - t|$. In the CrSb/2, 4 QL TI heterostructure, the strong hybridization interaction (t) between the top and bottom surfaces of the ultrathin topological insulator produces a large energy gap[9], resulting in a neglect of the average exchange field induced by the CrSb layer. As a consequence, the transport properties are dominated by the intrinsic magnetization of CrSb, yielding a single-step hysteresis loop. In contrast, in the CrSb/7, 12 QL TI system, the increased thickness of the TI effectively decouples the top and bottom surfaces[9], enabling the CrSb layer to couple predominantly with the top surface. In this case, the interfacial exchange field ($M_{\mathrm{ex}}^{\mathrm{AM}}$) opens a magnetic gap in the surface states, which in turn contribute significantly to the transport signal. This leads to the emergence of a two-step hysteresis loop, reflecting the coexistence of CrSb magnetism and surface-state-mediated transport. The consistency between our theoretical model and experimental observations supports this interpretation.

The overall anomalous Hall conductivity can be broadly divided into contributions from collinear-broken CrSb and interfacial exchange coupling:

$$\sigma_{xy}^{\text{Total}} = \sigma_{xy}^{\text{CrSb}} + \sigma_{xy}^{\text{ex}} \tag{8}$$

Following the Kubo formula, the intrinsic anomalous Hall conductance can be expressed as the integral of the Berry curvature of all occupied states within the Brillouin Zone[10,11]:

$$\sigma_{xy}^{\text{AHE}} = -\frac{2\pi e^2}{h}\sum_{a}\int \frac{d^3\mathbf{k}}{(2\pi)^3} f_a(\mathbf{k})\Omega_a(\mathbf{k}) \tag{9}$$

$$\Omega_a(\mathbf{k}) = \frac{2ih^2}{(2\pi)^2}\sum_{a\neq b}\frac{\langle \mathbf{k}, a|\hat{v}_x|\mathbf{k}, b\rangle\langle \mathbf{k}, b|\hat{v}_y|\mathbf{k}, a\rangle}{(E_a(\mathbf{k}) - E_b(\mathbf{k}))^2}$$

where h, e, and $f_a(\mathbf{k})$ are Planck's constant, electron charge, and distribution of the occupied states, respectively. $\Omega_a(\mathbf{k})$ is Berry curvature in **k** space. $\hat{v}_x$ and $\hat{v}_y$ are the velocity operators along the x and y directions. while $\langle \mathbf{k}, a|$ ($\langle \mathbf{k}, b|$) and $E_a(\mathbf{k})$ ($E_b(\mathbf{k})$) denote the eigenvectors and eigenvalues of the Hamiltonian, respectively. When the strong hybridization interaction (t) dominates σ_{xy}^{ex} contributes almost no conductivity signal. In contrast, if dominated by the interfacial exchange field ($M_{\text{ex}}^{\text{AM}}$), M_{ex} modifies the Berry curvature $\Omega_a^{\text{ex}}(\mathbf{k})$ at the interface, thus affecting the σ_{xy}^{ex} part. The interfacial exchange coupling ($M_{\text{ex}}^{\text{AM}}$) is generated by the coupling of the exchange field produced by CrSb with the topological surface states. In this case, the changes in exchange coupling only partially modify the shape of the overall anomalous Hall transport, while the overall transport is still determined by CrSb.

Section 10. Confirmation of the Fermi level of topological insulator $(Bi_{1-x}Sb_x)_2Te_3$.

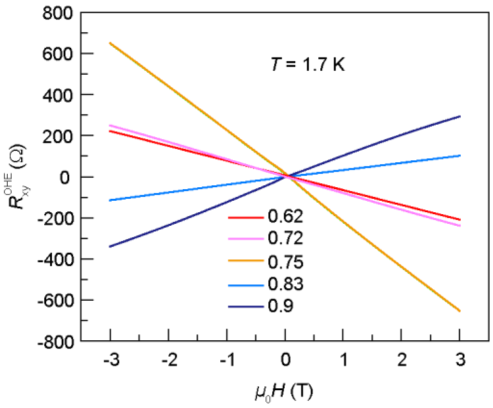

Figure S8. Hall resistance of $(Bi_{1-x}Sb_x)_2Te_3$ (x = 0.62, 0.72, 0.75, 0.83, and 0.90) thin films.

Table S1. Summary of carrier type of $(Bi_{1-x}Sb_x)_2Te_3$ with different Sb-to-Bi ratios.

Sb content x	Slope	Carrier type
0.62	Negative	N
0.72	Negative	N
0.75	Negative	N
0.83	Positive	P
0.90	Positive	P

To confirm the Fermi level and Dirac point positions of $(Bi_{1-x}Sb_x)_2Te_3$ thin films, we also grew a series of $(Bi_{1-x}Sb_x)_2Te_3$ (x = 0.62, 0.72, 0.75, 0.83, and 0.90) samples without CrSb and carried out Hall measurements on these samples. As shown in Fig. S8, all samples exhibit ordinary Hall resistance without any signature of AHE, indicating the absence of intrinsic ferromagnetism in the TI layer. This observation also highlights that the magnetic ordering in the TI layer is induced by the adjacent CrSb layer in the CrSb/TI heterostructures displaying the two-step Hall profiles. Furthermore, the slopes of the ordinary Hall effect and the corresponding carrier types for each sample are summarized in Table S1. Notably, a transition in the TI carrier type from N-type to P-type occurs within the Sb doping concentration range of 0.75–0.83, suggesting that the Dirac point lies approximately within this composition range. Leveraging this feature, we can achieve tunable interfacial spin textures in the CrSb/TI heterostructure by modulating the carrier type in the TI layer.

Section 11. Hybrid anomalous Hall resistance in the CrSb/$(Bi_{1-x}Sb_x)_2Te_3$ heterostructure with different TI carrier types.

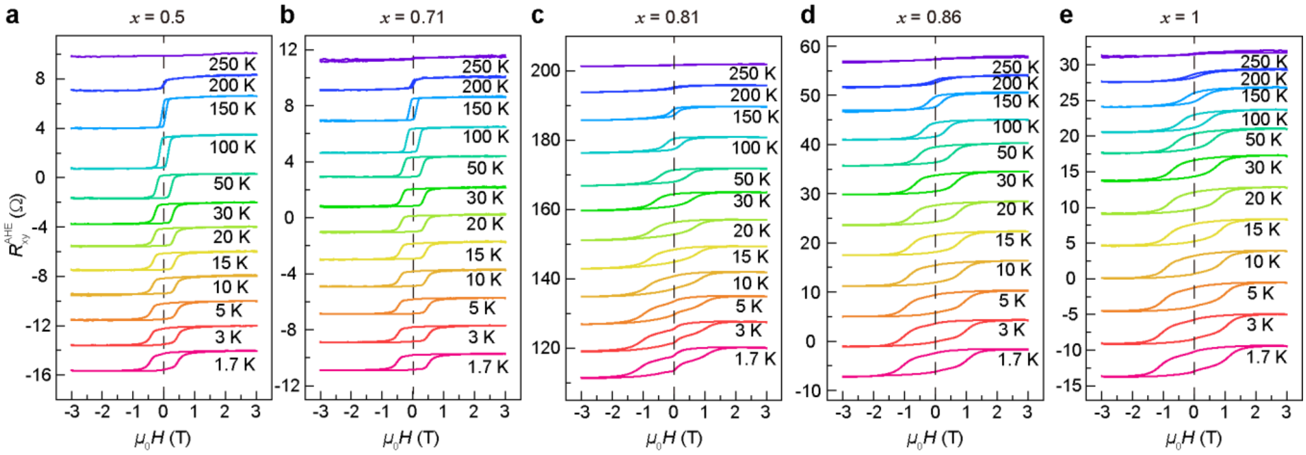

Figure S9. Temperature-dependent anomalous Hall resistance in the CrSb/$(Bi_{1-x}Sb_x)_2Te_3$ for various *x*-values. a-e, The evolution of the hybrid anomalous Hall resistance of 2.4 nm CrSb/7 QL $(Bi_{1-x}Sb_x)_2Te_3$ for x = 0.5, 0.71, 0.81, 0.86, and 1, from 1.7 K to 250 K.

Figure S9 shows the anomalous Hall hysteresis loops of 2.4 nm CrSb/7 QL $(Bi_{1-x}Sb_x)_2Te_3$ (x = 0.5, 0.71, 0.81, 0.86, and 1) heterostructures over the temperature range from 1.7 K to 250 K. At low temperatures (below 20 K), as the Sb concentration in the TI layer increases, a pronounced double-step anomalous Hall feature emerges at x = 0.81. This behavior reflects the presence of multiple magnetic components and indicates that the interfacial exchange coupling is significantly enhanced when the Fermi level of the TI is tuned near the Dirac point. At elevated temperatures, the anomalous Hall signals evolve into conventional ferromagnetic-like hysteresis, with the coercive field increasing monotonically as a function of Sb doping. This trend is attributed to strain effects arising from lattice mismatch at the CrSb/TI interface, which modify the interfacial spin texture. These observations are consistent with the results summarized in Fig. 3 in the main text.

Section 12. Reproducibility and statistics for CrSb/$(Bi_{1-x}Sb_x)_2Te_3$

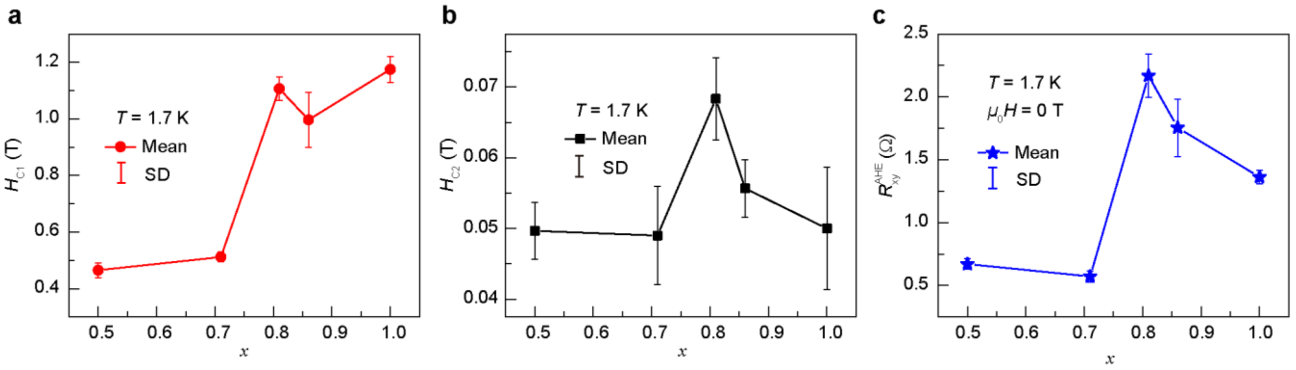

Figure S10. The average value with standard deviation for H_{c1}, H_{c2}, and zero-field R_{xy}^{AHE} based on 2.4 nm CrSb/7 QL $(Bi_{1-x}Sb_x)_2Te_3$. Mean is the average of H_{c1}, H_{c2}, and zero-field R_{xy}^{AHE} on three different areas, and SD is the standard deviation.

We performed magneto-transport measurements on three different areas of our wafer-scale samples and captured the corresponding H_{c1}, H_{c2}, and zero-field R_{xy}^{AHE}. Furthermore, we calculated the average value with the standard deviation, plotted in Fig. S10. The trend is consistent with Fig. 3 in the main text.

Section 13. The modified Kondorsky-type model.

To understand the magnetic evolution dynamics for angular dependence, the following total Kondorsky-type model can be established by[12]:

$$H_c = \frac{H_c(0°)(N_A + N_x)\cos(\varphi)}{N_z\sin(\varphi)^2 + (N_A + N_x)\cos(\varphi)^2} \tag{10}$$

where the parameters H_c, $N_i(i = x, y, z)$, N_A, and φ represent coercivity, demagnetizing factors, effect of magnetocrystalline anisotropy ($N_A = \frac{H_A}{M_S}$, H_A and M_S represent anisotropy field and saturation magnetization), and the angle between magnetic field and z-direction, respectively. In our thin films, the thickness of CrSb is fixed at 2.4 nm, and the easy axis is the z-direction. This means that the demagnetization effect along the z-direction N_z is about 1, and both N_x and N_y can be neglected. Therefore, we can simplify the above equation:

$$H_c = \frac{H_c(0°)N_A\cos(\varphi)}{\sin(\varphi)^2 + N_A\cos(\varphi)^2} \tag{11}$$

In our CrSb/TI heterostructure, the combined effects of strain and exchange coupling induce a tilt in the magnetization direction of Cr away from the normal axis. Moreover, due to the presence of exchange coupling mechanisms, it is challenging to directly analyze such a composite coercivity. Therefore, we decompose the coercivity of CrSb (H_{c1}) to facilitate a more detailed investigation, and a modified angle (φ_0) can be introduced into the above equation to account for both effects by:

$$H_{c1} = \frac{H_{c1}(0°)N_A\cos(\varphi + \varphi_0)}{\sin(\varphi + \varphi_0)^2 + N_A\cos(\varphi + \varphi_0)^2} \tag{12}$$

Fig. 4c in the main text presents an excellent agreement between the modified Kondorsky-type model and the experimental data. The non-zero fitting angle φ_0 further indicates that the strain effect induces a tilting of the Cr magnetic moments. Considering the presence of the tilt angle, fitting the data between 0° and 80° may ensure the validity of the model.

Section 14. Modified K-model fitting under variable temperatures

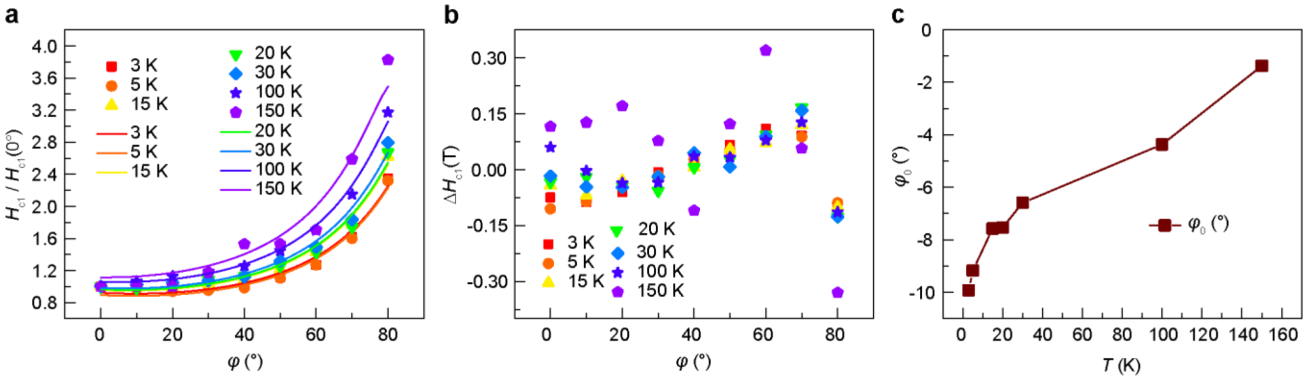

Figure S11. Modified K-model fitting of 2.4 nm CrSb/7 QL $(Bi_{0.19}Sb_{0.81})_2Te_3$ at variable temperatures. a, Modified K-model fitting from 3 to 150 K; the dots and lines denote the experimental and fitted results, respectively. **b**, The difference between the model and the experiment results. **c**, temperature-dependent fitted tile angles φ_0.

Figure S11a shows the fitted H_{c1} values using the modified K-model at 3-150 K, which aligns well with the experimental results. The tilt angle is approximately between -9.9° and -1.3°. However, we must emphasize that this tilt angle is more like an effective tilt angle determined by a combination of strain, interface coupling, and other effects. Meanwhile, we obtained the corresponding residuals (Fig. S11b), which are almost all within the range of approximately ±0.12 T. These results suggest that introducing a tilt angle into the modified K-model can reflect the magnetic dynamics of CrSb with broken collinear spin texture. The trend of the fitted tilt angle with temperature is summarized in Fig. S11c. The overall trend is that the magnitude of the tilt angle gradually decreases and approaches 0 with increasing temperature. Two different values of slope are observed below and above ~20 K, which seems to suggest different rates of decrease in the magnitude of the tilt angle, in agreement with the transition from two-step to single AHE around 20 K.

Section 15. Comparison of the classical K-model, modified K-model, and S-W model.

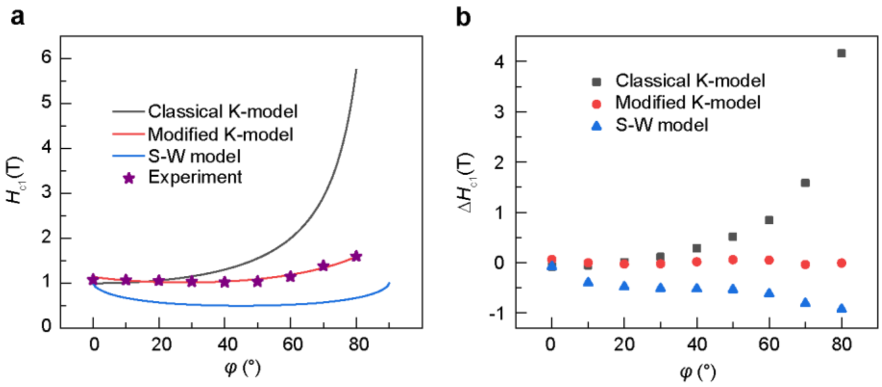

Figure S12. Comparison of the classical K-model, modified K-model, and S-W model based on H_{c1}. a, The classical K-model, modified K-model, and S-W model. **b**, The residuals between three models and the experiment results.

To confirm the existence of a reasonable tilt angle, we further compare the classical K-model, modified K-model, and S-W model. As shown in Fig. S12a, in contrast to the classical K-model and S-W model, the modified K-model enables the best fitting with experiments. We further extracted the residuals (ΔH_{c1}), shown in Fig. S12b. The ΔH_{c1} of the modified K-model is almost negligible, compared to that of the classical K-model and S-W model, significantly deviating from zero. These results suggest that the existence of the tilt angle is reasonable.

Section 16. Derivation of the effective Stoner-Wohlfarth model.

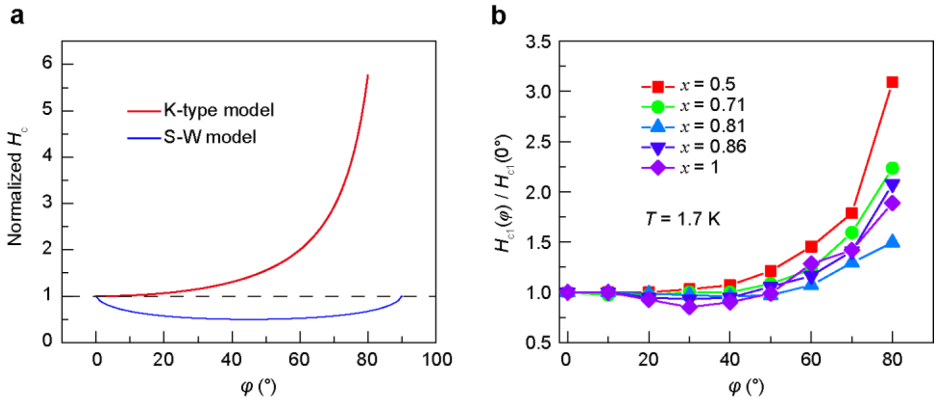

Figure S13. Comparison of models and experimental data. a, Computed normalized H_c based on classical Kondorsky[13] $(1/cos\varphi)$ and S-W model. **b**, Experimental angular-dependent coercivity H_{c1} of 2.4 nm CrSb/7 QL $(Bi_{1-x}Sb_x)_2Te_3$ (x = 0.5, 0.71, 0.81, 0.86, and 1) extracted from the measured anomalous Hall transport data.

To understand that the stronger exchange coupling is beneficial to stabilize altermagnetic order, we can build the total energy E including anisotropy, Zeeman energy, and exchange coupling terms in our heterostructure by:

$$E = K_1 sin^2\theta_1 + K_2 sin^2\theta_2 + M_1\mu_0 Hcos(\theta_1 - \varphi) + M_2\mu_0 Hcos(\theta_2 - \varphi) + J_{ex}cos(\theta_1 - \theta_2) \quad (13)$$

where $K_1(K_2)$, $M_1(M_2)$, and J_{ex} represent anisotropy, magnetization, and exchange coupling parameters, respectively. $\theta_1(\theta_2)$ is the angle between magnetization and easy axis, and $\mu_0 H$ is the applied magnetic field. To obtain the lowest energy state, we can obtain the first gradient of E for θ_1 and θ_2.

$$\frac{\partial E}{\partial \theta_1} = K_1 2sin\theta_1 cos\theta_1 + M_1\mu_0 Hsin(\theta_1 - \varphi) + J_{ex}sin(\theta_1 - \theta_2) = 0 \quad (14)$$

$$\frac{\partial E}{\partial \theta_2} = K_2 2sin\theta_2 cos\theta_2 + M_2\mu_0 Hsin(\theta_2 - \varphi) - J_{ex}sin(\theta_1 - \theta_2) = 0 \quad (15)$$

As the external magnetic field is gradually swept, a sudden transition emerges in the local minimum solution of the governing equations, indicating the emergence of magnetization reversal. The critical field at which this instability occurs corresponds to the coercive field. Here, we assume the coupling constant between Cr atoms to be J_{Cr}, with J_{ex} ranging from 0 to J_{Cr}. As the interfacial coupling strengthens, J_{ex} approaches J_{Cr}. In the limiting case where $J_{ex} \approx J_{Cr}$, the Cr-induced

magnetic ordering nearly aligns with that of the intrinsic Cr magnetization, indicating $\theta_1 \approx \theta_2 \approx \theta_{\mathrm{Cr}}$. Under such circumstances, we obtain:

$$\frac{\partial E}{\partial \theta_{\mathrm{Cr}}} = (K_1 + K_2)2sin\theta_{\mathrm{Cr}}cos\theta_{\mathrm{Cr}} + (M_1 + M_2)\mu_0 Hsin(\theta_{\mathrm{Cr}} - \varphi) = 0 \quad (16)$$

Notably, the effective Stoner–Wohlfarth (S–W) model[14] ($\propto (cos^{2/3}\varphi + sin^{2/3}\varphi)^{-3/2}$) for single domain can be obtained by this equation. Therefore, as the exchange field strengthens, the Cr-induced magnetic phase on TI increasingly aligns with the intrinsic magnetization direction of Cr. When the exchange field is sufficiently strong, the exchange coupling tends to align the induced magnetic phase at the TI surface more closely with the intrinsic magnetic phase of CrSb, resembling a single-domain behavior as described by the Stoner-Wohlfarth (S-W) model. This implies that stronger exchange coupling leads to CrSb exhibiting angle-dependent magnetization dynamics that more closely follow the S-W model, resulting in a relatively smaller variation in its magnetic ordering. This is consistent with our experimental observations. As shown in Fig. S13b, when the Fermi level of the topological insulator approaches the Dirac point (e.g., x = 0.81), the strengthened interfacial exchange coupling leads to a notably reduced variation in the altermagnetic configuration, indicating its enhanced robustness.

Section 17. Ruling out the current-induced heating effect.

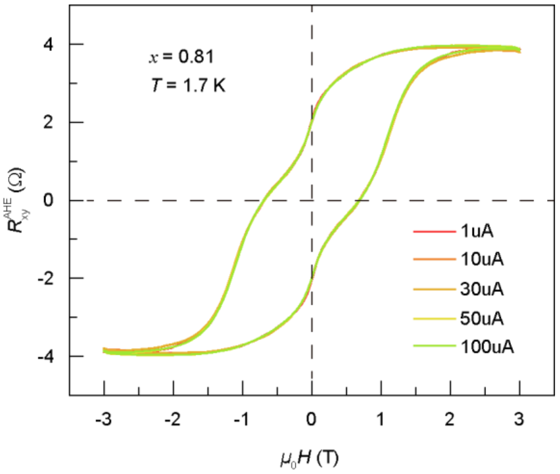

Figure S14. Transport signal of 2.4 nm CrSb/7 QL $(Bi_{0.19}Sb_{0.81})_2Te_3$ under different currents.

To rule out current-induced heating effects, we carried out magneto-transport measurements on the 2.4 nm CrSb/7 QL $(Bi_{0.19}Sb_{0.81})_2Te_3$ sample under various excitation currents I_{ac} = 1, 10, 30, 50, and 100 μA. As shown in Fig. S14, the two-step shape almost overlaps, which substantiates that the transport signal is independent on the amplitude of the excitation current.

References

1 Gibaud, A., Chebil, M. & Beuvier, T. X-ray reflectivity. *Surf. Sci. Techniq.*, 191-216 (2013).

2 Fan, Q. A new method of calculating interplanar spacing: the position-factor method. *Appl. Crystallogr.* **45**, 1303-1308 (2012).

3 Polesya, S. *et al.* Structural and magnetic properties of CrSb compounds: NiAs structure. *J Phys-Condens. Mat.* **24**, 036004 (2011).

4 Aota, S. & Tanaka, M. Epitaxial growth and transport properties of a metallic altermagnet CrSb on a GaAs (001) substrate. *Phys. Rev. Mater.* **9**, 074410 (2025).

5 DeGrave, J. P., Liang, D. & Jin, S. A general method to measure the Hall effect in nanowires: Examples of FeS_2 and MnSi. *Nano lett.* **13**, 2704-2709 (2013).

6 Luo, W. & Qi, X.-L. Massive Dirac surface states in topological insulator/magnetic insulator heterostructures. *Phys. Rev. B* **87**, 085431 (2013).

7 Lee, A. T., Han, M. J. & Park, K. Magnetic proximity effect and spin-orbital texture at the Bi_2Se_3/EuS interface. *Phys. Rev. B* **90**, 155103 (2014).

8 Zhang, F., Kane, C. L. & Mele, E. J. Surface state magnetization and chiral edge states on topological insulators. *Phys. Rev. Lett.* **110**, 046404 (2013).

9 Zhang, T., Ha, J., Levy, N., Kuk, Y. & Stroscio, J. Electric-field tuning of the surface band structure of topological insulator Sb_2Te_3 thin films. *Phys. Rev. Lett.* **111**, 056803 (2013).

10 Nagaosa, N., Sinova, J., Onoda, S., MacDonald, A. H. & Ong, N. P. Anomalous Hall effect. *Rev. Mod. Phys.* **82**, 1539 (2010).

11 Xiao, D., Chang, M.-C. & Niu, Q. Berry phase effects on electronic properties. *Rev. Mod. Phys.* **82**, 1959 (2010).

12 Suponev, N., Grechishkin, R., Lyakhova, M. & Pushkar, Y. E. Angular dependence of coercive field in (Sm, Zr)(Co, Cu, Fe) z alloys. *J Magn. Magn. Mater.* **157**, 376-377 (1996).

13 Cebollada, F., Rossignol, M., Givord, D., Villas-Boas, V. & González, J. Angular dependence of coercivity in Nd-Fe-B sintered magnets: Proof that coherent rotation is not involved. *Phys. Rev. B* **52**, 13511 (1995).

14 Tannous, C. & Gieraltowski, J. The Stoner–Wohlfarth model of ferromagnetism. *Eur. J. Phys.* **29**, 475 (2008).
