# Supplementary material for: Tailoring topological altermagnetic spin texture via interfacial exchange coupling in quasi-2D CrSb/(Bi, Sb)2Te3 thin film
Source: Nat Commun. 2026 Apr 17;17:5350. doi: 10.1038/s41467-026-72021-7 (PMC13272750; doi:10.1038/s41467-026-72021-7)
Supplement: Supplementary file 2 — Transparent Peer Review file [file 41467_2026_72021_MOESM2_ESM.pdf]

nature portfolio

Peer Review File

Tailoring topological altermagnetic spin texture via interfacial exchange coupling in quasi-2D CrSb/(Bi, Sb)2Te3 thin film

Corresponding Author: Dr Peng Chen

This file contains all reviewer reports in order by version, followed by all author rebuttals in order by version.

Version 0:

Reviewer comments:

Reviewer #1

(Remarks to the Author)
This manuscript reports pronounced and tunable anomalous Hall effects (AHE) in quasi-2D CrSb/(Bi_{1−x}Sb_x)_2Te_3 epitaxial heterostructures. The central claims are: (i) a double-step AHE emerging in thin CrSb coupled to a thicker TI layer, interpreted as the coexistence of strain-broken altermagnet (AM) order (high-coercivity branch $Hc1$) and interfacial exchange–driven topological contribution (low-coercivity branch $Hc2$); (ii) the thickness contrast between 2 QL and 7 QL TI (hybridization t strong vs. weak) controls whether the AHE is single- or double-stepped; (iii) Fermi-level tuning by Sb content peaks the effect near $x≈0.81$ where the TI Fermi level approaches the Dirac point; and (iv) the angle-dependent coercivity is consistent with a modified Kondorsky-type model with a finite tilt angle $φ0$. These elements are clearly stated in the conclusions/methods and in the angle-dependence analysis of the main text and Supplementary Sections 5–9. The novelty lies in placing an altermagnet × topological-insulator interface into the quasi-2D limit and systematically linking symmetry-broken AM order, TI-surface-state hybridization, and dual-coercivity AHE—with the further claim that tuning t and EF in the TI offers a route to engineer “topological altermagnetic” responses. However, the current structure–band–transport loop is not yet closed: several key inferences rely on indirect transport signatures without element-resolved or band-resolved corroboration; the theory stops short of a Berry-curvature/Kubo semi-quantitative closure; and statistics/controls need to rule out exchange-spring/two-phase and extrinsic AHE alternatives. I recommend Major Revision.
1、Provide cross-section HR-STEM/HAADF with EDS/EELS plus element-specific magnetism (XMCD or PNR) on the same specimens to exclude Cr-rich ferromagnetic inclusions, interfacial metallization, or exchange-spring two-phase scenarios as alternative origins of the second coercive branch.

2、Supply ARPES/SARPES over a 2–10 QL series to quantify the Dirac-state gap and spin texture, fit an exponential t(QL), and correlate it with the extracted Hc2(QL) from transport to convert the 2-vs-7 QL contrast into a systematic band–transport linkage.

3、Demonstrate gate-tunable reversibility on the same device: map Hc2(Vg) and zero-field Rxy^AHE(Vg) across the Dirac-point crossing to reproduce the composition-tuned peak without batch or disorder confounds.

4、Close the theory–experiment loop by computing Berry curvature and Kubo sigma_xy(EF; t, Mex) for your effective model and comparing magnitude/sign and trends versus x and temperature; report fitted parameters with uncertainties.

5、Perform an intrinsic/extrinsic AHE scaling analysis: plot sigma_xy^AHE versus rho_xx (or sigma_xx) over devices and temperatures with error bars and sample counts; demonstrate that skew and side-jump channels cannot reproduce the observed double-step evolution.

6、Test model identifiability in the angle dependence: fit both the modified Kondorsky and single-domain Stoner–Wohlfarth forms to 0–360° datasets, report residuals/AIC/BIC, and verify that the tilt angle is uniquely required rather than a surrogate for multi-phase switching.

7、Quantify the link strain → tilt angle by extracting in-plane/out-of-plane lattice constants (HRXRD/RSM) and assembling a statistical correlation (with confidence intervals) between strain-tensor components and the fitted tilt parameter.

8、Document the ordinary Hall subtraction pipeline (linear/polynomial/two-carrier) and show robustness of the zero-field

AHE amplitude/sign to the chosen method; include residual analysis of raw Rxy(H) for representative compositions and thicknesses.

9、Bound planar Hall and AMR cross-terms via symmetry tests (antisymmetrization, device rotation) and geometry flips; provide an upper-bound fraction relative to the zero-field AHE to ensure the double step is not a mixing artifact.

10、Provide current-amplitude/frequency dependence (e.g., 1–100 microamp) of Hc1, Hc2, and zero-field AHE to rule out self-heating or contact nonlinearity in the lock-in measurements.

11、Report device statistics and reproducibility for each x and QL: N, yield, mean ± s.d. for Hc1, Hc2, rho_xx, rho_yx; include minor-loop/cycling tests to assess history/training effects.

12、Test whether the x≈0.81 peak could arise from multi-band transport alone by two-carrier fits to OHE segments and by showing that such fits cannot generate a genuine double-step AHE without interfacial exchange.

13、Provide vector or microscope-MOKE imaging versus temperature and field to visualize whether the two AHE components correspond to distinct domain/phase processes and to map them onto the two coercive fields extracted from transport.

14、In the 2 QL limit, consider and bound vertical shunting or edge-channel contributions arising from top–bottom surface hybridization; use multi-terminal layouts or bar-flip checks to confirm geometry-independent AHE conclusions.

15、Define consistently the “transition temperature(s)” (criteria from dM/dH or dRxy^AHE/dH), and show magnetometry- and transport-derived scales agree across cycles without training effects.

16、Expand the thickness series beyond 2 and 7 QL (e.g., 3–6, 8–10 QL) to establish a continuous t(QL) trend and connect it to the temperature window where the double step persists or vanishes.

17、Provide a strain–coercivity scatter (e.g., Hc1 versus extracted strain with uncertainties) to substantiate the claim of strain-broken collinear altermagnetism beyond qualitative description.

18、Show full-range angle scans (0–360°) at multiple temperatures with fit residuals; report confidence intervals for the tilt angle and any additional anisotropy parameters, and discuss their physical bounds.

19、Present uncertainty propagation from device geometry and contact resistance into Hc1, Hc2, and sigma_xy; confirm that the reported large change in zero-field AHE with composition remains well beyond systematic error bars.

20、Discuss operational limits and integration: to what extent can the quasi-2D altermagnetic signal survive toward device-relevant temperatures and/or with thicker CrSb; outline a pathway (materials or electrostatic control) to move the demonstrated effects closer to practical operating windows.

Reviewer #2

(Remarks to the Author)
The authors investigate the anomalous Hall effect (AHE) in quasi-2D CrSb (2.4 nm)/$(Bi_{1-x}Sb_x)_2Te_3$ heterostructures grown by MBE. They demonstrate that growth on the topological insulator induces tensile strain in the CrSb layer due to lattice mismatch, which breaks the collinear altermagnetic spin symmetry, and produces pronunced AHE and tunable spin anisotropy. Magnetization measurements reveal two-step hysteresis loops in thicker TI layers, indicating the coexistence of strain modified altermagnetic order and interfacial exchange coupling. The AHE strength is shown to be tunable via both the strain and the Fermi-level position of the TI. An effective Hamiltonian model describes how exchange coupling between the altermagnetic order and the TI surface states gives rise to a hybrid AHE.

Overall, I find the paper solid and presenting significant results on quasi-2D interfaces between an altermagnet and a topological insulator. It highlights the crucial role of strain and exchange coupling in modifying the altermagnetic spin texture, but I think that a more direct experimental verification of the altermagnetic state in strained CrSb would further strengthen the work.

Version 1:

Reviewer comments:

Reviewer #1

(Remarks to the Author)
The author addressed the questions very well and I have no concern now and would like to recommend this paper to be publish in Nature Communications!

Reviewer #2

(Remarks to the Author)
I think the authors have addressed all reasonable experimental concerns raised by the reviewers, providing additional data and clarifications within the practical limitations of their system. While some direct measurements are not feasible due to sample constraints, the combination of transport data, structural characterization, and comparison with previous literature provides a coherent and convincing picture. Overall, the manuscript presents novel and significant results, and I consider it suitable for publication.

Reviewer #3

(Remarks to the Author)
This manuscript systematically investigates MBE-grown quasi-2D CrSb (2.4 nm)/$(Bi_{1-x}Sb_x)_2Te_3$ heterostructures, proposing that interfacial tensile strain breaks the collinear altermagnetic symmetry of CrSb to induce a pronounced anomalous Hall effect (AHE). By fine-tuning the topological insulator (TI) thickness and Sb doping ratio, the authors observe a remarkable transition from a single- to a two-step AHE hysteresis loop. Supported by an effective Hamiltonian and a modified Kondorsky model, this dual-component behavior is convincingly attributed to the coexistence of strain-broken intrinsic altermagnetism and interfacial topological surface state exchange coupling. Although the work is outstanding in its experimental design and comprehensiveness of data, I find that several critical issues remain regarding the "closing of the loop" between macroscopic transport phenomena and the microscopic magnetic structure/theoretical models. Therefore, I recommend major revisions to fully address the following critical issues.
1. Considering that conducting precise first-principles calculations on alloy superlattices is rather complex, could the authors provide a qualitative, mechanism-focused physical discussion (based on crystal field theory or a simplified spin-orbit coupling Hamiltonian) on the causal link between interfacial strain and the emergence of net magnetic moment? Specifically, how does in-plane tensile strain alter specific Cr-Sb bond lengths or angles, thereby preferentially breaking the initially compensated g-wave altermagnetic symmetry rather than causing other forms of magnetic disorder?
2. The effective Hamiltonian model in the main text only considers the out-of-plane component of the exchange field from CrSb. Given that the modified Kondorsky model reveals a tilted effective moment, could the authors briefly discuss the influence of an in-plane exchange field component on the Hamiltonian eigenstates and the resulting AHE, to ensure consistency between the two theoretical analyses?
3. The authors introduced the equivalent tilt angle in the modified Kondorsky model to perfectly fit the angle-dependent H_{c1} data, showing fitting results between 3 K and 15 K in their rebuttal. Given that the equivalent tilt angle is considered a comprehensive manifestation of strain and interfacial coupling, could the authors systematically analyze and discuss the specific evolutionary trend of this fitted the equivalent tilt angle parameter as the temperature increases? Does this trend align with the thermal attenuation of the interfacial coupling strength as it approaches 20 K (the critical temperature where the two-step behavior disappears)?
4. Since the system contains a strain-broken altermagnetic dominant phase (H_{c1}) and a weaker interfacial topological surface phase (H_{c2}), this constitutes a typical magnetically coupled interface. In the current minor-loop cycling tests, the reversals of these two phases appear quite independent. Could the authors discuss whether any exchange bias effects (i.e., a horizontal shift of the magnetic hysteresis loop along the magnetic field axis) caused by the interaction between these two phases were observed under field-cooling conditions or specific bias magnetic fields?
5. Currently, the main evidence for interfacial topological exchange coupling is centered on the low-coercivity branch (H_{c2}) in the transverse anomalous Hall resistance (R_{xy}). To further corroborate this coupling effect from a complementary perspective, could the authors examine and present whether there are any subtle anomalous features in the temperature or magnetic-field-dependent curves of the longitudinal resistance (R_{xx}) near the transition temperature (~20 K) that correspond to the decoupling of the surface states or magnetic scattering?

Version 2:

Reviewer comments:

Reviewer #3

(Remarks to the Author)
The authors have thoroughly addressed all the raised concerns, and the manuscript is now suitable for publication.

Open Access This Peer Review File is licensed under a Creative Commons Attribution 4.0 International License, which permits use, sharing, adaptation, distribution and reproduction in any medium or format, as long as you give appropriate credit to the original author(s) and the source, provide a link to the Creative Commons license, and indicate if changes were made.
In cases where reviewers are anonymous, credit should be given to 'Anonymous Referee' and the source.
The images or other third party material in this Peer Review File are included in the article’s Creative Commons license, unless indicated otherwise in a credit line to the material. If material is not included in the article’s Creative Commons license and your intended use is not permitted by statutory regulation or exceeds the permitted use, you will need to obtain permission directly from the copyright holder.
To view a copy of this license, visit https://creativecommons.org/licenses/by/4.0/

Tailoring topological altermagnetic spin texture via interfacial exchange coupling in quasi-2D $CrSb/(Bi, Sb)_2Te_3$ thin film

Peng Chen[1], Josep Ingla-Aynés[1], Chen Chang[2], Eugene Park[2], Don Heiman[1,3], Frances M. Ross[2], Hang Chi[4,5,6], & Jagadeesh S. Moodera[1,7]

[1]*Francis Bitter Magnet Laboratory, Plasma Science and Fusion Center, Massachusetts Institute of Technology, Cambridge, Massachusetts 02139, USA*

[2]*Department of Materials Science and Engineering, Massachusetts Institute of Technology, Cambridge, Massachusetts 02139, USAs*

[3]*Department of Physics, Northeastern University, Boston, Massachusetts 02115, USA*

[4]*Department of Physics, University of Ottawa, Ottawa, Ontario K1N 6N5, Canada*

[5]*School of Electrical Engineering and Computer Science, University of Ottawa, Ottawa, Ontario K1N 6N5, Canada*

[6]*Nexus for Quantum Technologies, University of Ottawa, Ottawa, Ontario K1N 6N5, Canada*

[7]*Department of Physics, Massachusetts Institute of Technology, Cambridge, Massachusetts 02139, USA*

Response to Reviewer 1 Comments:

General comments:

This manuscript reports pronounced and tunable anomalous Hall effects (AHE) in quasi-2D CrSb/(Bi_Sb_x)_2Te_3 epitaxial heterostructures. The central claims are: (i) a double-step AHE emerging in thin CrSb coupled to a thicker TI layer, interpreted as the coexistence of strain-broken altermagnet (AM) order (high-coercivity branch $Hc1$) and interfacial exchange–driven topological contribution (low-coercivity branch $Hc2$); (ii) the thickness contrast between 2 QL and 7 QL TI (hybridization t strong vs. weak) controls whether the AHE is single- or double-stepped; (iii) Fermi-level tuning by Sb content peaks the effect near $x \approx 0.81$ where the TI Fermi level approaches the Dirac point; and (iv) the angle-dependent coercivity is consistent with a modified Kondorsky-type model with a finite tilt angle $\varphi 0$. These elements are clearly stated in the conclusions/methods and in the angle-dependence analysis of the main text and Supplementary Sections 5–9. The novelty lies in placing an altermagnet × topological-insulator interface into the quasi-2D limit and systematically linking symmetry-broken AM order, TI-surface-state hybridization, and dual-coercivity AHE—with the further claim that tuning t and EF in the TI offers a route to engineer "topological altermagnetic" responses. However, the current structure–band–transport loop is not yet closed: several key inferences rely on indirect transport signatures without element-resolved or band-resolved corroboration; the theory stops short of a Berry-curvature/Kubo semi-quantitative closure; and statistics/controls need to rule out exchange-spring/two-phase and extrinsic AHE alternatives. I recommend Major Revision.

Response: We sincerely thank Reviewer 1 for the recognition of our work in the novelty between altermagnet and topology. In response to your comments, we made revisions in the manuscript and also provided relevant data to further clarify the statements as follows:

1. We provided the cross-section HR-STEM with the EDS result (Suppl. Section 2 to Comment 1).

2. We supplied the TI thickness-dependent magneto-transport result (Suppl. Section 6 to Comment 2).

3. We demonstrated the gating device as a future direction (Page 8 to Comment 3).

4. We built the Berry curvature equation about conductance to explain the single and two-step hysteresis loops (Suppl. Section 8 to Comments 4 and 5).

5. We performed an intrinsic/extrinsic AHE scaling analysis (Comment 5).

6. We fitted the classical K-model, the modified Kondorsky, and the single-domain Stoner–Wohlfarth to support the validity of the modified Kondorsky model to explain our findings (Suppl. Section 14 and Page 10 to Comments 6 and 7).

7. We described the ordinary Hall extraction pipeline (Suppl. Section 4 and Page 5 to Comments 8 and 9).

8. We provided current-dependent anomalous Hall resistance (Suppl. Section 16 and Page 12 to Comment 10).

9. We reported device statistics and reproducibility (Suppl. Section 11 and Page 8 to Comment 11).

10. We confirmed geometry-independent AHE using multi-terminal layouts and bar-flip (Suppl. Section 5 and Page 5 to Comment 14).

11. We define the “transition temperature(s)” in the main text (Page 6 to Comment 15).

12. We attributed our results to strain-broken collinear altermagnetism using XRD peak shift and the corresponding hysteresis loop (Page 8 to Comment 17).

13. We fitted the data at multiple temperatures with fit residuals based on the modified K-model (Suppl. Section 13 and Page 10 to Comment 18).

14. We explained how to eliminate the longitudinal signal due to device geometry (Suppl. Section 4 to Comment 19).

15. We outlined a possible pathway to move the demonstrated effects closer to practical operating windows (Page 10 to Comment 20).

Additionally, we have also made necessary modifications in the manuscript to further enhance the readability andensure that our work is more accessible to the general audience in the condensed matter physics research community.

Comment 1: *Provide cross-section HR-STEM/HAADF with EDS/EELS plus element-specific magnetism (XMCD or PNR) on the same specimens to exclude Cr-rich ferromagnetic inclusions, interfacial metallization, or exchange-spring two-phase scenarios as alternative origins of the second coercive branch.*

Response to comment 1:

We performed HR-STEM and EDS analysis on thicker CrSb/TI. HR-STEM displayed the TI and CrSb layers above and below the interface, shown in Figs. R1a-b. EDS shows a relatively uniform distribution of Cr confined almost entirely within the CrSb layer above the interface (Fig. R1c), which rules out Cr-rich regions and interfacial metallization. Furthermore, combined with our RHEED images (Fig. R1d, a reproduction of Fig. 1b), which show only a single set of diffraction streaks for $(Bi,Sb)_2Te_3$ (BST) and CrSb, and XRD showing only peaks corresponding to BST, CrSb, and the substrate (Fig. R1e, a reproduction of Fig. 1c), we can exclude the presence of hard/soft two-phase exchange-spring behavior.

XMCD can provide the average magnetic moment through the orbital information (e.g, $L_{2,3}$ of Cr) of magnetic elements[1]. In our CrSb/TI samples, there is only one magnetic element (Cr), and XMCD would

detect only the moment signal of Cr. It is not suitable to exclude Cr-rich, interfacial metallization, or an exchange-spring two-phase case. For PNR investigation, since our CrSb layer is only 2.4 nm, following the previously reported results[2], it is impossible to obtain a reliable spin asymmetry signal with a big uncertainty.

Nevertheless, based on our comprehensive HR-STEM, RHEED, and XRD data, we can exclude Cr-rich, interfacial metallization, and the hard/soft two-phase exchange-spring case. Accordingly, we added HR-STEM and EDS to the **Suppl. Section 2, Figure S2,** and revised the corresponding contents in the **main text (Page 4)**.

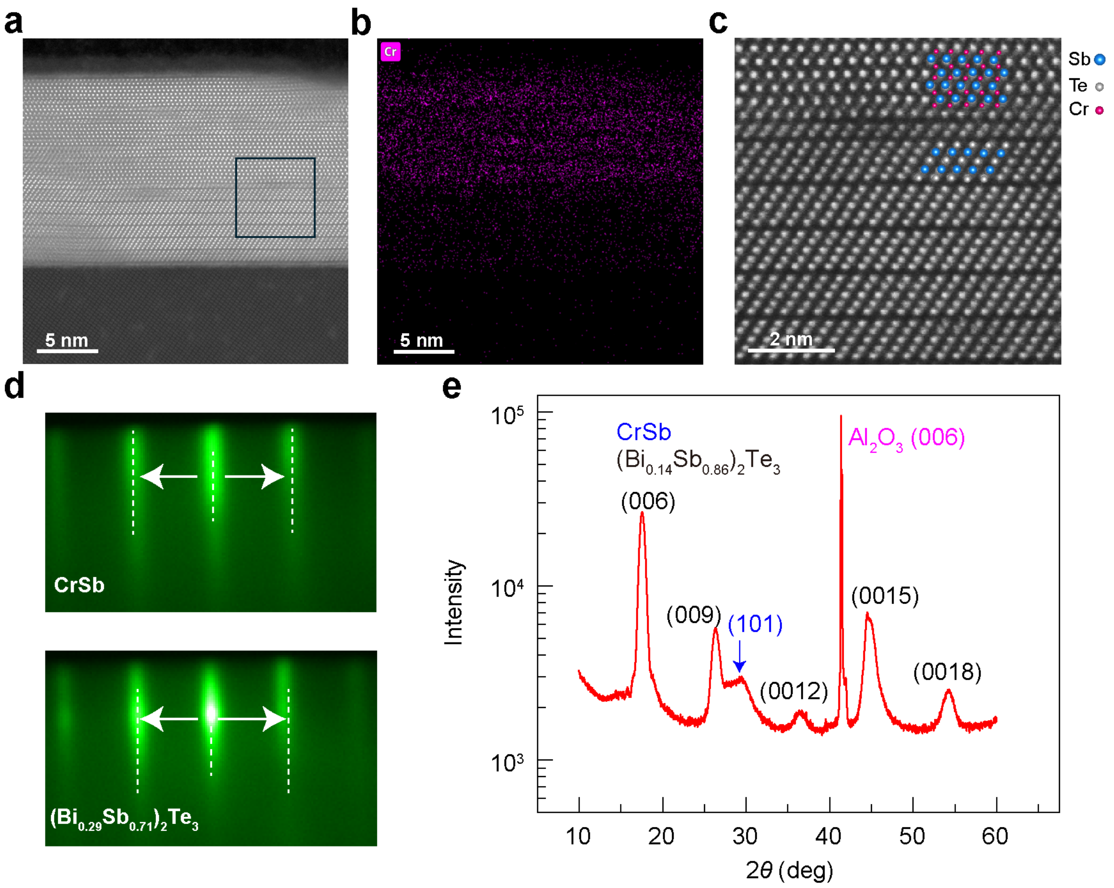

Figure R1. Sample structure characterizations of CrSb/TI heterostructures. a, Cross-sectional HR-STEM image on thicker CrSb/Sb_2Te_3. **b,** Distribution of Cr in this image. **c,** Enlarged view of the atomic arrangement in the indicated box in **a**. Panels (**a-c**) are now in **Fig. S2a-c**. **d,** RHEED patterns of CrSb and $(Bi_{0.29}Sb_{0.71})_2Te_3$. The sharp stripe patterns indicate two-dimensional growth, and the distance between arrows indicates the reciprocal lattice unit. **e,** XRD analysis of CrSb/$(Bi_{0.14}Sb_{0.86})_2Te_3$ shows that all peaks are accounted for by the expected phases.

Comment 2: *Supply ARPES/SARPES over a 2–10 QL series to quantify the Dirac-state gap and spin texture, fit an exponential t(QL), and correlate it with the extracted Hc2(QL) from transport to convert the 2-vs-7 QL contrast into a systematic band–transport linkage.*

Response to comment 2:

To address this issue, we further grew the 2.4 nm CrSb/4 QL TI and 2.4 nm CrSb/12 QL TI, and carried out the magneto-transport measurement. As shown in Fig. R2.1, as the thickness of TI increases from 2 QL to 4 QL, the coercivity due to strain gradually increases to 0.780 T, while no obvious two-step behavior is observed. With further increase to 7 QL and 12 QL, the transport results show a distinct two-step line shape, which is attributed to the altermagnetic order and topological surface states broken by the strain effect. Furthermore, we extracted the H_{c2}. As shown in Fig. R2.2, H_{c2} of the 2.4 nm CrSb/2, 4 QL TI cases is 0, and H_{c2} of the 2.4 nm CrSb/7, 12 QL TI cases is around 0.060-0.065T.

In our CrSb/TI heterostructures, the interface is buried, and the sample surface is covered with a 1-3 nm AlO_x capping layer. Normal ARPES is a highly surface-sensitive technique[3], presenting challenges including charge accumulation issues and difficulty in detecting the interface for our samples. Nevertheless, previously *in-situ* ARPES and STM on topological insulators (TIs) have been investigated[4,5]. As reported there[4,5], direct evidence from ARPES and STM experiments demonstrates that approximately 4 QL is a critical thickness at which hybridization between the top and bottom surfaces leads to a reduction in the energy gap and significant changes in topological properties[4,5]. Our transport results are consistent with the reported ARPES and STM results[4,5]. Accordingly, we added additional transport results in the **Suppl. Section 6** and revised the **main text (Pages 5 and 7)**.

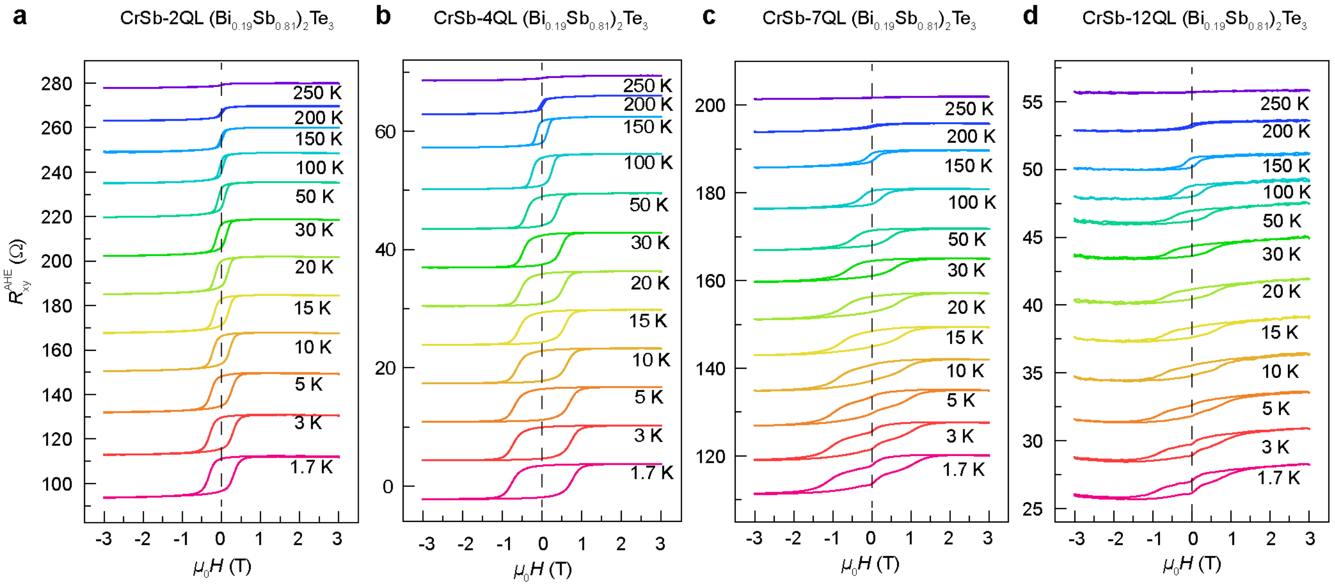

Figure R2.1. Temperature-dependent anomalous Hall resistance. a-d, Temperature evolution of anomalous Hall resistance of 2.4 nm CrSb with different thickness $(Bi_{0.19}Sb_{0.81})_2Te_3$ (2, 4, 7, 12 QL) from 1.7 K to 250 K.

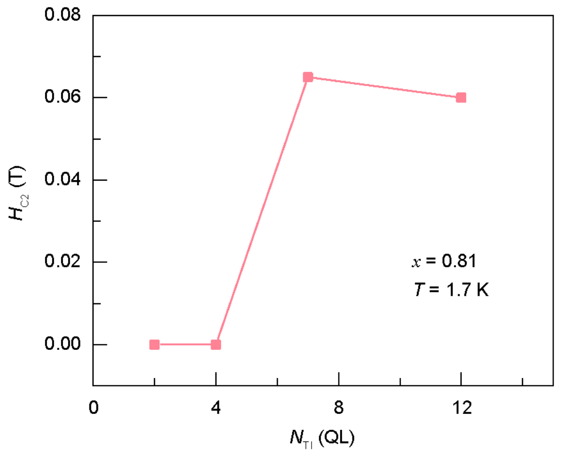

Figure R2.2. H_{c2} versus the thickness of TI with x = 0.81 at 1.7 K.

Comment 3: *Demonstrate gate-tunable reversibility on the same device: map Hc2(Vg) and zero-field Rxy^AHE(Vg) across the Dirac-point crossing to reproduce the composition-tuned peak without batch or disorder confounds.*

Response to comment 3:

The discussion of gating in the original manuscript was intended only as a forward-looking illustration of potential device architectures. In the present work, our primary objective is to demonstrate that chemical tuning via Sb doping is sufficient to stably and systematically shift the Fermi level of TI across the Dirac point, thereby producing a more than 300% change in R_{xy}. This composition-controlled approach already captures the essential physical picture, showing that the anomalous Hall effect becomes significantly enhanced when the Fermi level crosses the Dirac point.

To avoid possible misunderstanding, we have clarified in the **revised manuscript (Page 8)** that the gating discussion is presented solely as a “future direction to be explored.”

Comment 4: *Close the theory–experiment loop by computing Berry curvature and Kubo sigma_xy(EF; t, Mex) for your effective model and comparing magnitude/sign and trends versus x and temperature; report fitted parameters with uncertainties.*

Response to comment 4:

The overall anomalous Hall conductivity can be broadly divided into contributions from collinear-broken CrSb and interfacial exchange coupling:

$$\sigma_{xy}^{Total} = \sigma_{xy}^{CrSb} + \sigma_{xy}^{ex}$$

Following the Kubo formula, the intrinsic anomalous Hall conductance can be expressed as the integral of the Berry curvature of all occupied states within the Brillouin Zone[6,7]:

$$\sigma_{xy}^{\mathrm{AHE}} = -\frac{2\pi e^2}{h}\sum_{a}\int \frac{d^3\boldsymbol{k}}{(2\pi)^3} f_a(\boldsymbol{k})\boldsymbol{\Omega}_a(\boldsymbol{k})$$

$$\boldsymbol{\Omega}_a(\boldsymbol{k}) = \frac{2ih^2}{(2\pi)^2}\sum_{a\neq b}\frac{\langle \boldsymbol{k},a|\hat{v}_x|\boldsymbol{k},b\rangle\langle \boldsymbol{k},b|\hat{v}_y|\boldsymbol{k},a\rangle}{(E_a(\boldsymbol{k}) - E_b(\boldsymbol{k}))^2}$$

Where h, e, and $f_a(\boldsymbol{k})$ are Planck's constant, electron charge, and distribution of the occupied states, respectively. $\boldsymbol{\Omega}_a(\boldsymbol{k})$ is Berry curvature in $\boldsymbol{k}$ space. $\hat{v}_x$ and $\hat{v}_y$ are the velocity operators along the x and y directions. while $\langle \boldsymbol{k},a|$ ($\langle \boldsymbol{k},b|$) and $E_a(\boldsymbol{k})$ ($E_b(\boldsymbol{k})$) denote the eigenvectors and eigenvalues of the Hamiltonian, respectively. Our transport measurements show that CrSb dominates the transverse transport signal of the entire system. When we change the Bi/Sb ratio, all of the transport measurements exhibit a clear hysteresis loop. This means that the magnetic states of the CrSb material keep collinearity broken. In addition, controlling the Bi/Sb ratio also modulates the interfacial exchange coupling (M_{ex}^{AM}) through modified Fermi level of TI. M_{ex}^{AM}, in turn, modifies the Berry curvature $\boldsymbol{\Omega}_a^{ex}(\boldsymbol{k})$ at the interface, thus affecting the σ_{xy}^{ex} part. However, the interfacial exchange coupling (M_{ex}^{AM}) is generated by the coupling of the exchange field produced by CrSb with the topological surface states. In this case, the changes in exchange coupling only partially modify the shape of the overall anomalous Hall transport, while the overall transport is still determined by CrSb. As shown in our Fig. 3a, the polarity of all AHE is positive and dominated by CrSb, and the two-step shapes are different.

In the system with varying Bi/Sb ratios, it is out of the scope of the present manuscript to theoretically construct supercells with the Bi and Sb sites ideally matching that of the experimental Bi/Sb ratio, as it poses significant computational challenges for density functional theoretical simulations. Nevertheless, we explained the physics governing the sign of AHE as a function of x using well-established Berry curvature formulism and the effective Hamilton model. Accordingly, we added the discussion to **Suppl. Section 8**.

Comment 5: *Perform an intrinsic/extrinsic AHE scaling analysis: plot sigma_xy^AHE versus rho_xx (or sigma_xx) over devices and temperatures with error bars and sample counts; demonstrate that skew and side-jump channels cannot reproduce the observed double-step evolution.*

Response to comment 5:

Here, we took 2 different R_{xy} channels to carry out the magneto-transport measurement. We plotted σ_{xy} and σ_{xx} (Figs. R3a-b) of different temperatures, and this nonlinear correlation excludes the dominant influence of skew scattering[6]. The error bar is less than 10%. Meanwhile, when we changed the Bi/Sb ratio, the anomalous Hall resistance at zero field changed by 300% from x = 0.71 to 0.81 (Fig. R3c, re-captured from Fig. 3f in the main text), indicating a modification of the intrinsic properties related to the band structure of the topological insulator[8]. This modulation of band-related intrinsic characteristics originates from the interfacial topological altermagnetic texture.

On the other hand, skew scattering or side-jump are extrinsic mechanisms associated with impurity scattering. Two-step hysteresis loops are usually produced by two magnetic subsystems[9]. Here, we assume that the transport behavior of the two magnetic systems comes from skew scattering or side-jump, which means they are not expected to exhibit any direct dependence on the thickness of the adjacent layer (TI). However, our 2.4 nm CrSb/2 QL and 7 QL TI samples exhibit single and two-step hysteresis loops, respectively, ruling out the assumption that the two-step transport features originate from extrinsic scattering. Regarding the physical origin of the AHE in CrSb or magnetic TI material systems, extensive relevant literature also indicates that it arises from intrinsic Berry curvature[10,11]. Accordingly, we revised the corresponding **Suppl. Section 8**.

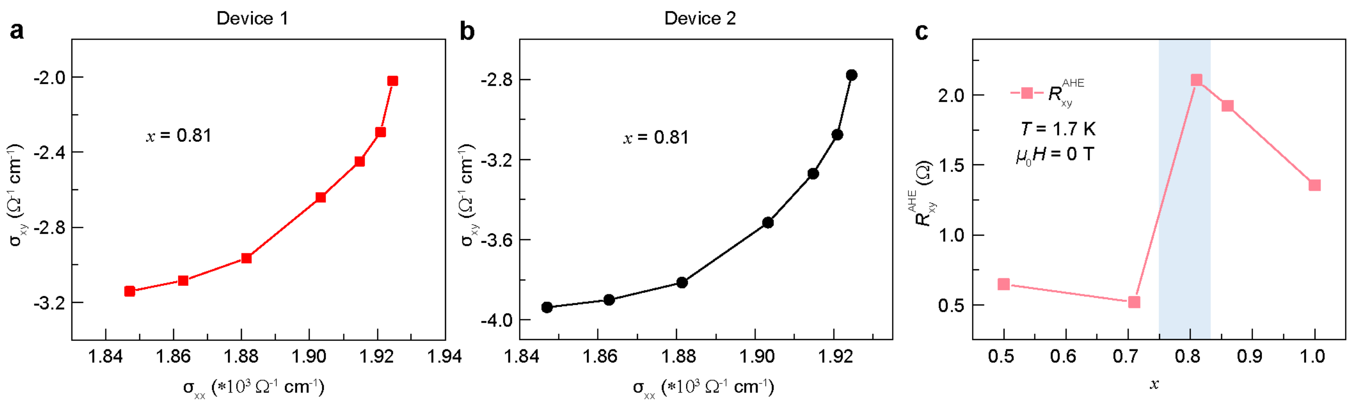

Figure R3. The magneto-transport measurements on CrSb/TI heterostructures. a-b, Conductance of CrSb/TI with x = 0.81 under different temperatures. c, Zero-field R_{xy}^{AHE} of CrSb/$(Bi_{1-x}Sb_x)_2Te_3$ extracted from measured anomalous Hall resistance at T = 1.7 K. As Sb increases from 0.71 to 0.81, zero-field R_{xy}^{AHE} increases about 300%.

Comment 6: *Test model identifiability in the angle dependence: fit both the modified Kondorsky and single-domain Stoner–Wohlfarth forms to 0–360° datasets, report residuals/AIC/BIC, and verify that the tilt angle is uniquely required rather than a surrogate for multi-phase switching.*

Response to comment 6:

For the classical K-model[12], the coercivity is proportional to $1/\cos\varphi$. According to the formula, when φ approaches 90°, the coercivity tends towards infinity. However, this is impossible in experiments. Therefore, the angle range for K-type and S-W models fitting is typically between 0° and 90°, as widely adopted in the literature[12,13]. Although we modified the K-type model, it is still under the classical K-model frame. Considering including the tilt angle in the modified K-model, fitting the data between 0° and 80° may ensure the validity of the model. As shown in Fig. R4a, the modified K-model can be fitted with the experiment results (one can notice the increasing trend for H_{c1} as the angle approaches 90°). Furthermore, Fig. R4b displayed the residuals (fitting data minus experiment result) from the modified K-model fluctuate around 0 T, while the residuals from the S-W model gradually move away from 0 T. The fitting results and residuals suggest that the induction of a tilt angle is reasonable. Accordingly, we added the residuals from the two models to the **Suppl. Section 14** and explained the angle range of fitting in the supplementary Section 14 and the **main text (Page 10)**.

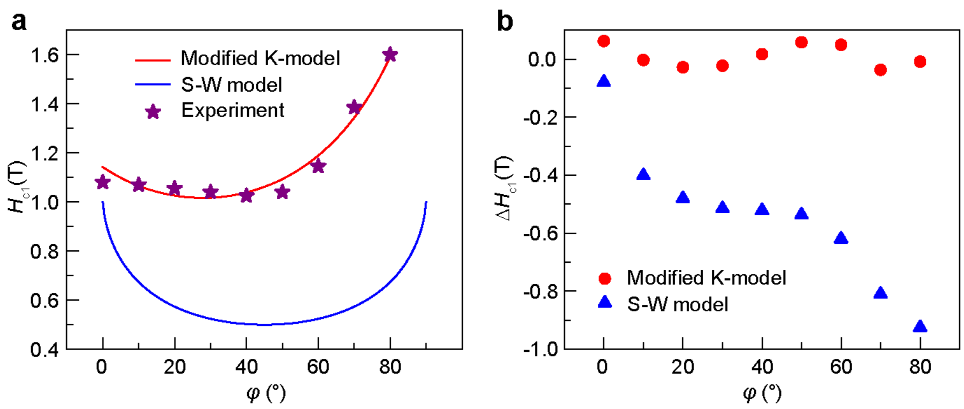

Figure R4. The comparison of the modified K-model and the S-W model based on H_{c1}. a, Modified K-model and S-W model fitting with experimental results. **b**, The residuals between the two models and the experiment results.

Comment 7: *Quantify the link strain → tilt angle by extracting in-plane/out-of-plane lattice constants (HRXRD/RSM) and assembling a statistical correlation (with confidence intervals) between strain-tensor components and the fitted tilt parameter.*

Response to comment 7:

As reported in the literature[14] and we already mentioned in the main text, the peak shift obtained through XRD can conveniently determine whether the strain is tensile or compressive. The XRD peak shift indicates that the in-plane lattice of CrSb is under tensile strain (Fig. R5a, re-capture from Fig. 1d in the main text), which is consistent with the ideal fully relaxed bulk in-plane lattice constant[15,16]. However, quantifying the

spin tilt angle caused by lattice mismatch in heterostructures using XRD is very challenging. Therefore, we must emphasize that the fitting spin tilt angle in our modified model is more like an equivalent tilt angle, combining strain and interface effects, etc. To further confirm the rationality of the tilt angle, we additionally fitted the classical K-model with a 0° tilt angle and obtained its residuals (Figs. R5b-c). The K-model with a 0° tilt angle showed poor fitting with very large residuals (maximum value ~ 4 T), which further demonstrates the necessity of the tilt angle. Accordingly, we added the classical K-model fitting to the **Suppl. Section 14** and revised the **main text (Page 10)**.

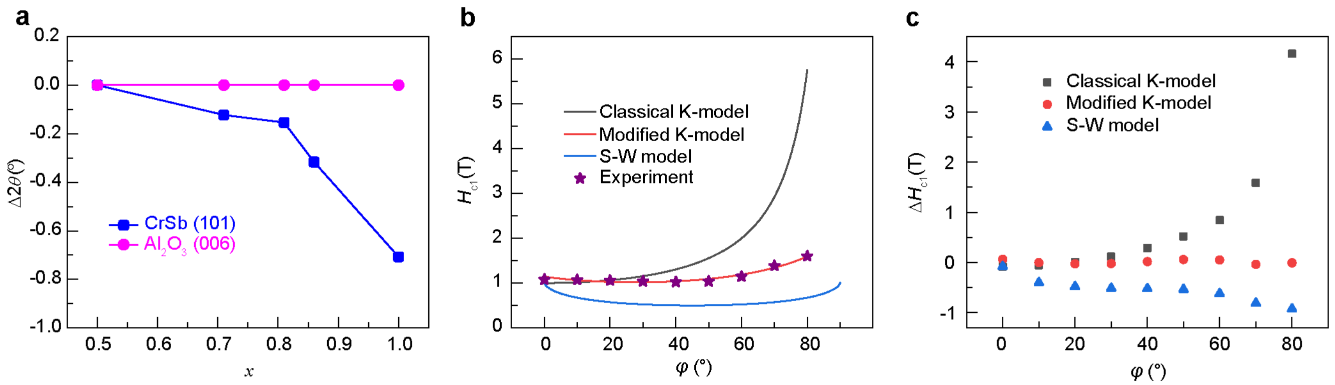

Figure R5. XRD peak shift and the comparison of the classical K-model, modified K-model, and S-W model based on H_{c1}. a, Shift of XRD peak position of Al_2O_3 (006) and CrSb (101) as a function of Sb content *x*. **b**, The classical K-model, modified K-model, and S-W model fitting with experimental results. **c**, The residuals between the three models and the experiment results.

Comment 8: *Document the ordinary Hall subtraction pipeline (linear/polynomial/two-carrier) and show robustness of the zero-field AHE amplitude/sign to the chosen method; include residual analysis of raw Rxy(H) for representative compositions and thicknesses.*

Response to comment 8:

The ordinary Hall was subtracted via linear fitting. In general, the Hall resistance can be expressed as $R_{xy} = R_{xy}^{\mathrm{OHE}} + R_{xy}^{\mathrm{AHE}} = R_0 \cdot \mu_0 H + R_s \cdot M_m$, R_0, R_s, and M_m represent ordinary Hall coefficient, anomalous Hall coefficient, and the uncompensated magnetization arising from the breaking of collinear altermagnetic order respectively. We take the 2.4 nm CrSb/ 7 QL $(Bi_{0.19}Sb_{81})_2Te_3$ sample as an example (Figs. R6a-c). In this case, we selected the two linear regions from 2.5 to 3 T and from -3 to -2.5 T and performed linear fits of R_{xy} versus magnetic field $\mu_0 H$ in each region. The slopes extracted from the two high-field linear segments are then averaged to obtain the ordinary Hall coefficient R_0. The anomalous Hall contribution is subsequently obtained as: $R_{xy}^{\mathrm{AHE}} = R_{xy} - R_0 \cdot \mu_0 H$. Accordingly, the AHE contribution can be captured in our manuscript. As shown in Fig. R6d (re-capture from Fig. 3a in our main text), it displayed the robust AHE sign. In fact,

the “residual” referred to by the reviewer is the anomalous Hall effect contribution. Its physical significance arises from the Hall response generated by the magnetic properties of the material. Accordingly, we added the ordinary Hall contribution extraction pipeline to the **Suppl. Section 4** and revised the **main text (Page 5)**.

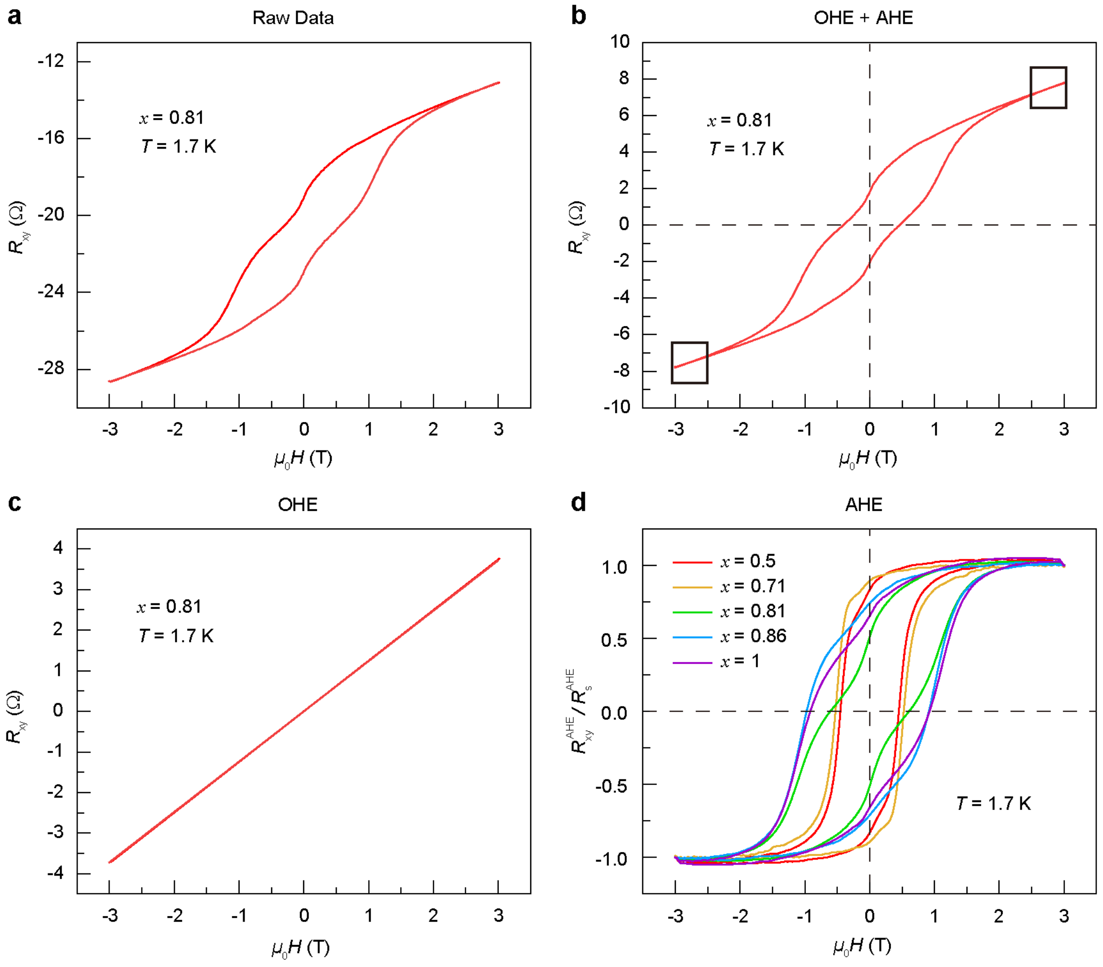

Figure R6. Extracted the ordinary Hall contribution of CrSb/TI. a, The raw data of 2.4 nm CrSb/7QL $(Bi_{0.19}Sb_{0.81})_2Te_3$. **b**, the Ordinary Hall effect (OHE) and AHE contribution after subtracting the longitudinal contribution. **c**, the OHE contribution via high magnetic field fitting marked in two black boxes of **b**. **d**, The normalized R_{xy}^{AHE} displays a hybrid anomalous Hall response in the CrSb/7 QL $(Bi_{1-x}Sb_x)_2Te_3$ (x = 0.5, 0.71, 0.81, 0.86, and 1) samples at T = 1.7 K.

Comment 9: *Bound planar Hall and AMR cross-terms via symmetry tests (antisymmetrization, device rotation) and geometry flips; provide an upper-bound fraction relative to the zero-field AHE to ensure the double step is not a mixing artifact.*

Response to comment 9:

In general, OHE and AHE measurements are performed with the magnetic field perpendicular to the sample surface (as in our case), resulting in magnetic field-dependent transport characteristics[17], shown in Fig. R7a. While PHE and AMR measurements involve a magnetic field within the plane of the current and depend on the angle between the magnetic field and the current[17], as demonstrated in Fig. R7b. Here, the longitudinal signal due to geometric misalignment is possible to induce to transverse signals as a background[18,19], but the Hall voltage is an odd function of the magnetic field and can thus be extracted from the transverse signal by antisymmetrization. The standard procedure[18,19] is to extract the antisymmetric component of the transverse signal using $R_{xy} = (R_{xy}^{Raw}(+\mu_0 H)\text{-}R_{xy}^{Raw}(\text{-}\mu_0 H))/2$, which includes OHE and AHE, as shown in Fig. R7c. Subtracting the ordinary Hall component from Fig. R7c (see comment 8) yields the two-step AHE contribution (Fig. R7d). This ensures that the result after antisymmetrization does not contain PHE and AMR components, and the double step is not a mixing artifact.

Meanwhile, the single hysteresis loop of the 2.4 nm CrSb/2 QL $(Bi_{0.19}Sb_{0.81})_2Te_3$ sample also ruled out the possibility that the double step was due to system or external artifacts. Accordingly, we emphasized how to eliminate the longitudinal transport signal from device geometric antisymmetrization in our ordinary Hall extraction process (**Suppl. Section 4**).

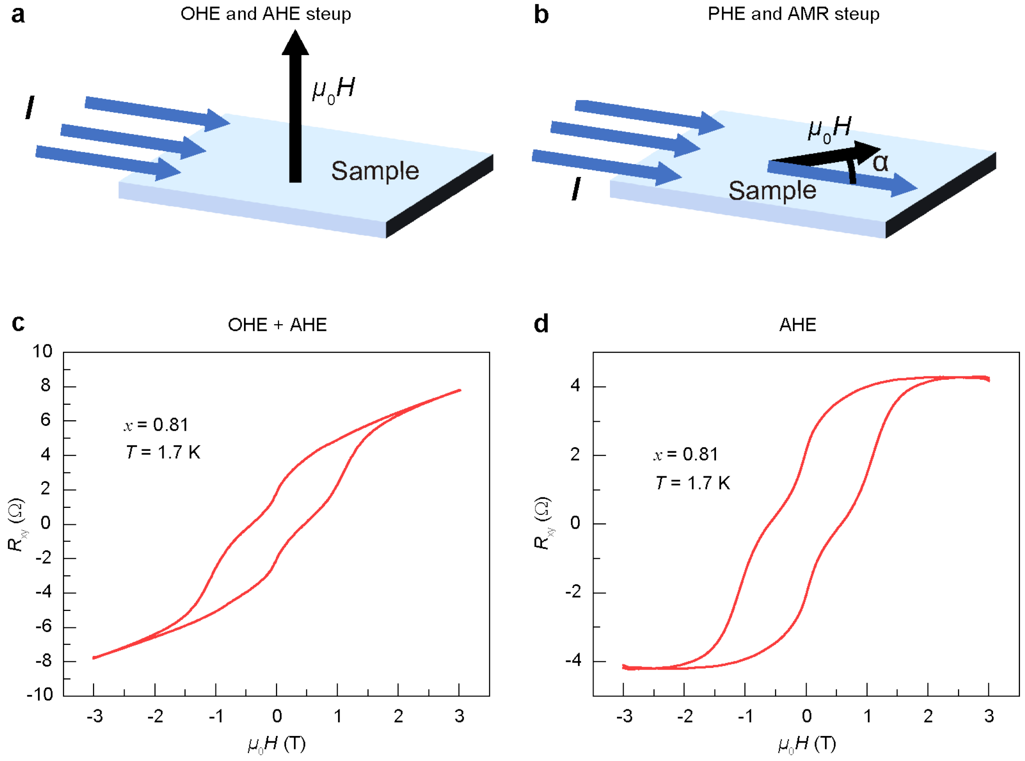

Figure R7. Extracted the two-step Hall contribution of CrSb/TI. a, The OHE and AHE measurement setup. b, The PHE and AMR measurement setup. c, the R_{xy} result after the antisymmetric process. d, The two-step AHE component.

Comment 10: *Provide current-amplitude/frequency dependence (e.g., 1–100 microamp) of Hc1, Hc2, and zero-field AHE to rule out self-heating or contact nonlinearity in the lock-in measurements.*

Response to comment 10:

To rule out self-heating or contact nonlinearity, we carried out the 2.4 nm CrSb/7 QL $(Bi_{0.19}Sb_{0.81})_2Te_3$ under currents I = 1, 10, 30, 50, and 100 μA. As shown in Fig. R8, the anomalous Hall resistance at different currents all show the same two-step behavior, and the curves overlap almost exactly, which means that the values of H_{c1}, H_{c2}, and zero-field anomalous Hall resistance are not caused by current heating. Accordingly, we added to the **Suppl. Section 16** and revised the **Method Section (Page 12)**.

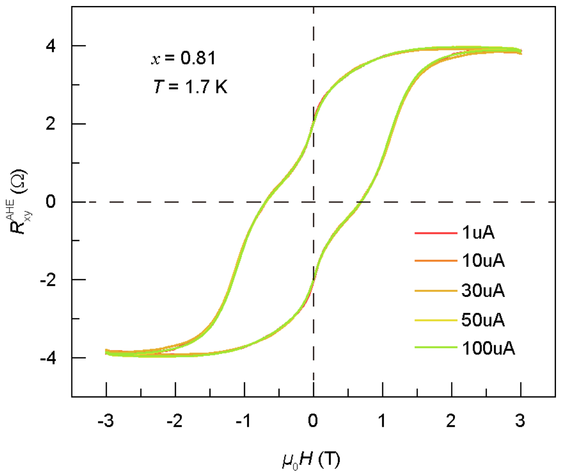

Figure R8. The two-step Hall measurement of CrSb/TI with x = 0.81 under different currents.

Comment 11: *Report device statistics and reproducibility for each x and QL: N, yield, mean ± s.d. for Hc1, Hc2, rho_xx, rho_yx; include minor-loop/cycling tests to assess history/training effects.*

Response to comment 11:

On the wafer, we selected three different regions and tested the corresponding transport signals. A two-step hysteresis loop was observed in both the 2.4nm CrSb / 7QL TI, while a single hysteresis loop was observed in the 2.4nm CrSb/2QL TI sample (e.g., see comment 14). We averaged the values of H_{c1}, H_{c1}, and R_{xy}^{AHE} (0 T) with the standard deviation, shown in Figs. R9a-c. The trends of H_{c1}, H_{c1}, and R_{xy}^{AHE} (0 T) are consistent with our observation described in the manuscript. The approximate range of R_{xx} was 0.4-2.4 kΩ. In addition, we performed five cyclic tests on the sample at x = 0.81, confirming the robustness of the two-step behavior without training effects, displayed in Fig. R9d. Accordingly, we added the results to the **Suppl. Section 11 and revised the main text (Page 8)**.

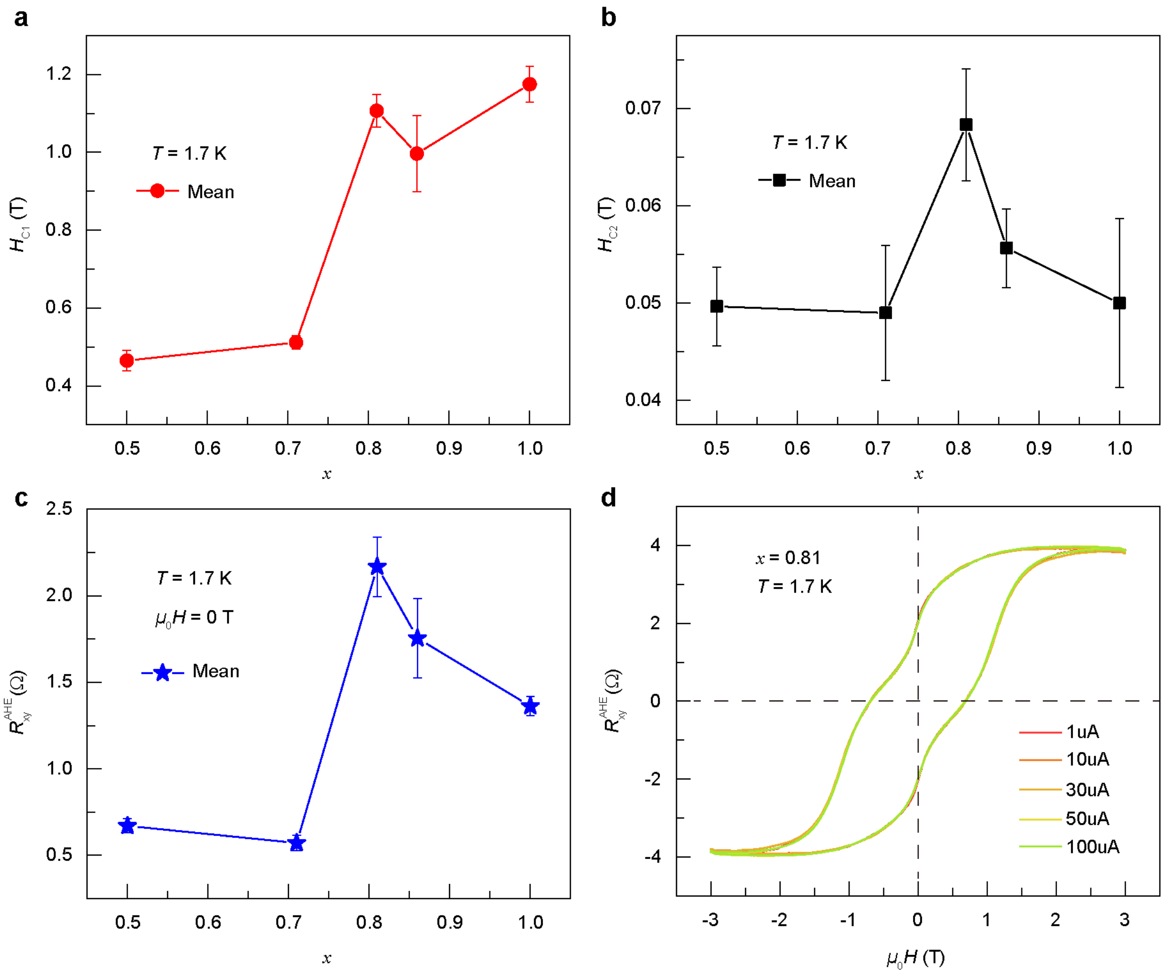

Figure R9. Repeatable measurements for CrSb/TI. a-c, The average value with standard deviation for H_{c1}, H_{c2}, and zero-field R_{xy}^{AHE} based on 2.4 nm CrSb/ 7 QL $(Bi_{1-x}Sb_x)_2Te_3$. d, five cyclic tests on the sample at $x = 0.81$.

Comment 12: *Test whether the x≈0.81 peak could arise from multi-band transport alone by two-carrier fits to OHE segments and by showing that such fits cannot generate a genuine double-step AHE without interfacial exchange.*

Response to comment 12:

The two-carrier model only produces shapes with two different overlapping slopes (Fig. R10a), but it cannot generate a two-step AHE signal with a hysteresis loop (Fig. R10b). This is because this model is related to the charge properties of the carriers, while the generation of AHE originates from the spin characteristics of the magnetic atoms.

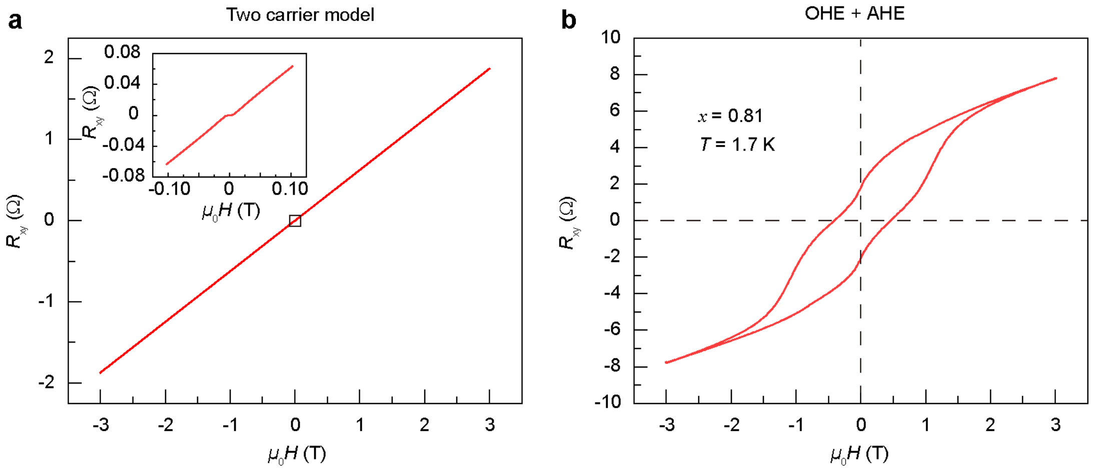

Figure R10. Two-carrier model fitting for CrSb/TI. a, two-carrier fitting result. **b**, the OHE and AHE components with double-peak.

Comment 13: *Provide vector or microscope-MOKE imaging versus temperature and field to visualize whether the two AHE components correspond to distinct domain/phase processes and to map them onto the two coercive fields extracted from transport.*

Response to comment 13:

Although MOKE can provide direct images of magnetic reversal, in our system, the interface is buried within the heterostructure and covered by a 1-3 nm AlO_x insulating layer, which not only affects the optical signal but may also render it ineffective[20]. In particular, the magnetic reversals at H_{c1} and H_{c2} are not instantaneous, and H_{c1} dominates the reversal, merging with H_{c2}, making it difficult to distinguish between the two components. Nevertheless, our additional thickness-dependent, temperature-dependent, and cyclic tests, as well as the Hamiltonian theoretical model, all support the validity of this two-step process.

Comment 14: *In the 2 QL limit, consider and bound vertical shunting or edge-channel contributions arising from top–bottom surface hybridization; use multi-terminal layouts or bar-flip checks to confirm geometry-independent AHE conclusions.*

Response to comment 14:

We carried out transport measurements on two different channels and the corresponding bar-flips (rotated by 180° for the two channels). As shown in Fig. R11, all of the results displayed a single hysteresis loop, which confirms geometry-independent AHE measurement in our samples. Accordingly, we added to the **Suppl. Section 5** and modified the **main text (Page 5)**.

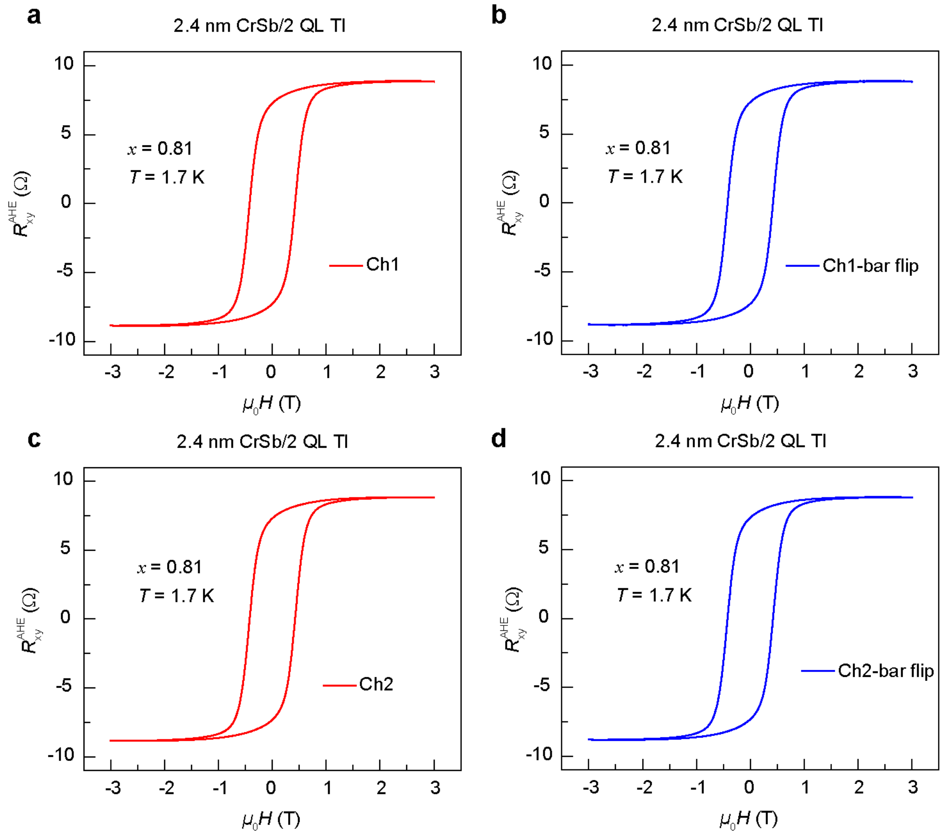

Figure R11. Magnetic field-dependent AHE based on channels 1, 1-bar flip (rotated by 180°), 2, and 2-bar flip (rotated by 180°) of 2.4 nm CrSb/2 QL $(Bi_{0.19}Sb_{0.81})_2Te_3$.

Comment 15: *Define consistently the "transition temperature(s)" (criteria from dM/dH or dRxy^AHE/dH), and show magnetometry- and transport-derived scales agree across cycles without training effects.*

Response to comment 15:

We have defined the transition temperature as the disappearance temperature of the coercive field. Accordingly, we revised the corresponding content as "In addition, the transition temperature (the disappeared temperature of the coercive field) of 2.4 nm CrSb/ 2QL $(Bi_{0.19}Sb_{0.81})_2Te_3$ (~250 K) is larger than that of 7.2 nm CrSb/ 2QL $(Bi_{0.19}Sb_{0.81})_2Te_3$ (50-100 K) (Supplementary section 4), indicating that the interfacial effects tend to dominate as the system approaches the 2D limit, leading to enhanced transition temperature". To observe the small hysteresis loop for magnetometry- and transport-derived scales, we also carried out the SQUID measurement at T = 200 K. As shown in Fig. R12, both SQUID and AHE display the corresponding small hysteresis loop. Accordingly, we defined the "transition temperature" in the **revised main text (Page 6)**.

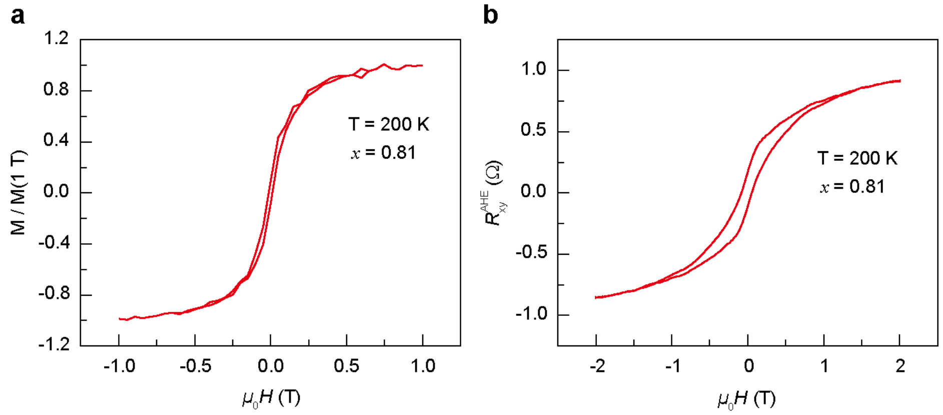

Figure R12. Magnetometry- and transport-dependent sample with x = 0.81 result at T = 200 K.

Comment 16: *Expand the thickness series beyond 2 and 7 QL (e.g., 3–6, 8–10 QL) to establish a continuous t(QL) trend and connect it to the temperature window where the double step persists or vanishes.*

Response to comment 16:

We further grew the 2.4 nm CrSb/4 QL TI and 2.4 nm CrSb/12 QL TI, and carried out the magneto-transport measurements. As reported in the literature, approximately 4 QL is the critical thickness at which hybridization between the top and bottom surfaces leads to a reduction in the energy gap. In our transport measurements, we observed an increase in coercivity to 0.78 T, but still no two-step behavior. In contrast, a two-step behavior was observed in both the 7 QL and 12 QL cases, where the temperature at which this unique characteristic disappears is approximately 15-20 K. At these thicknesses, the decoupling of the top and bottom surface states leads to enhanced AM/TI interfacial coupling, which is consistent with our Hamiltonian theoretical model. Accordingly, we added additional transport results to the **Suppl. Section 6** and revised the **main text (Pages 5 and 7).**

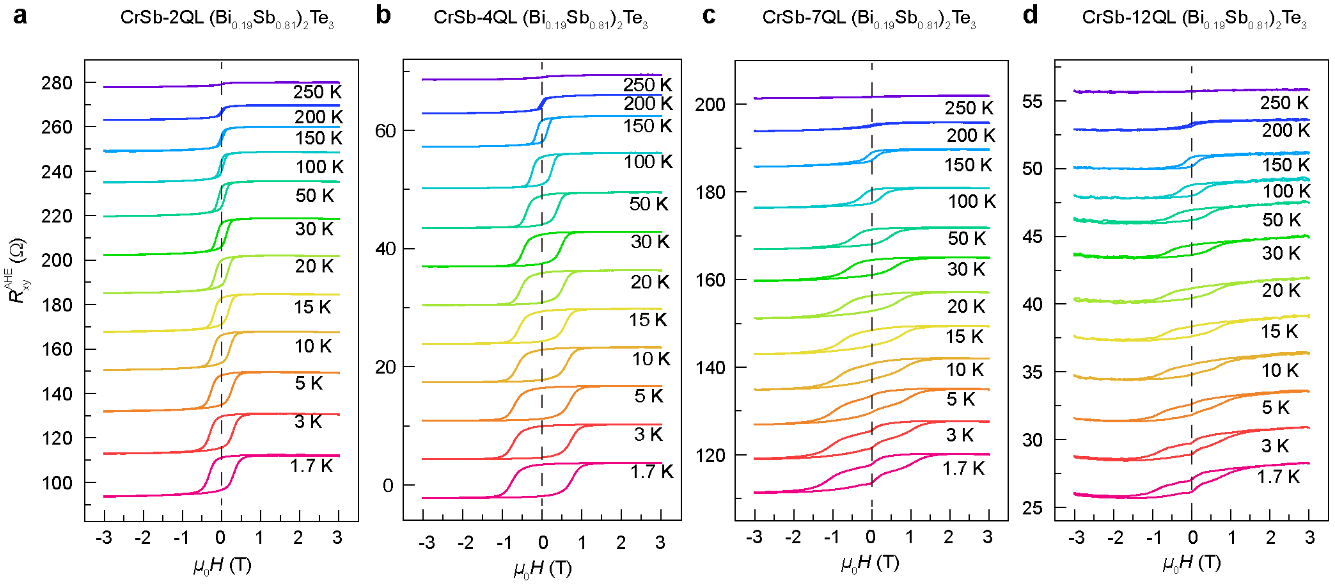

Figure R13. Temperature-dependent anomalous Hall resistance. a-d, Temperature evolution of anomalous Hall resistance of 2.4 nm CrSb with different thickness $(Bi_{0.19}Sb_{0.81})_2Te_3$ (2, 4, 7, 12 QL) from 1.7 K to 250 K.

Comment 17: *Provide a strain–coercivity scatter (e.g., Hc1 versus extracted strain with uncertainties) to substantiate the claim of strain-broken collinear altermagnetism beyond qualitative description.*

Response to comment 17:

There is a peak shift of XRD of CrSb with changing Sb/Bi ratio (Fig. R14a, re-captured from Fig. 1d), which means that the tensile strain of the CrSb in-plane lattice undergoes a relative change. Correspondingly, with the Sb/Bi ratio increasing, the coercivity (H_{c1}) displays the corresponding enhancement (Fig. R14b, re-captured from Fig. 3a). This supported the claim of strain-broken collinear altermagnetism. Accordingly, we further emphasize it in the **revised main text (Page 8)**.

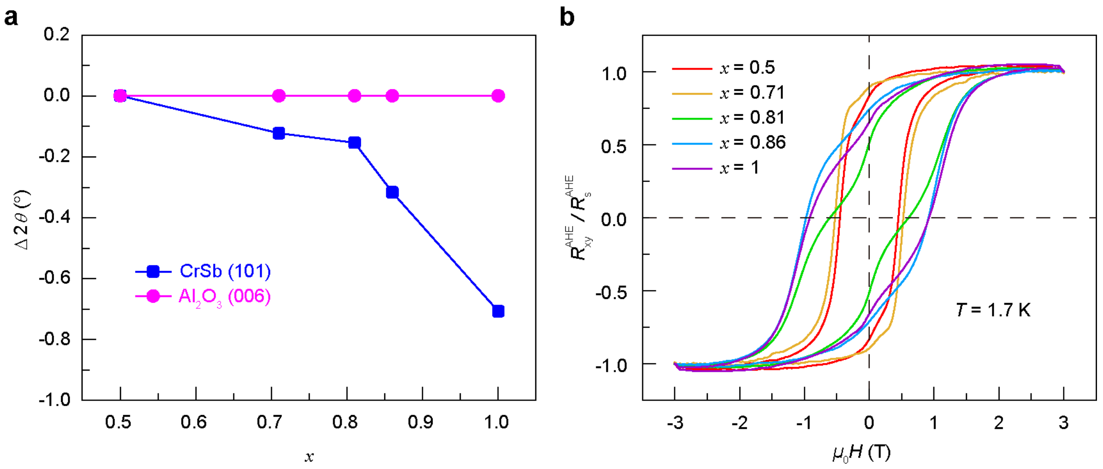

Figure R14. XRD peak shift and normalized anomalous Hall resistance. a, Shift of XRD peak position of Al_2O_3 (006) and CrSb (101) as a function of Sb content *x*. **b,** The normalized R_{xy}^{AHE} displays a hybrid anomalous Hall response in the CrSb/7 QL $(Bi_{1-x}Sb_x)_2Te_3$ (x = 0.5, 0.71, 0.81, 0.86, and 1) samples at T = 1.7 K.

Comment 18: *Show full-range angle scans (0–360°) at multiple temperatures with fit residuals; report confidence intervals for the tilt angle and any additional anisotropy parameters, and discuss their physical bounds.*

Response to comment 18:

As we mentioned in comment 6, the classic K-model predicts infinite coercivity at 90°, which is impossible experimentally. Therefore, we performed fitting in the 0°-80° range, which ensures the validity of the model due to the induction of tilt angle. We fitted H_{c1} at 3, 5, and 15 K (Fig. R15a), with the tilt angle approximately

between -9.9° and -7.5°. We attempted to change the tilt angle to obtain a confidence interval of approximately ±3°, with N_A ranging from 30 to 50. We also obtained the corresponding residuals (Fig. R15b), which were all within approximately ±0.11 T. However, we must emphasize that the fitting spin tilt angle in our modified model is more like an equivalent tilt angle, combining all strain, interface effects, etc. Accordingly, we added to the **Suppl. Section 13** and revised the **main text (Page 10)**.

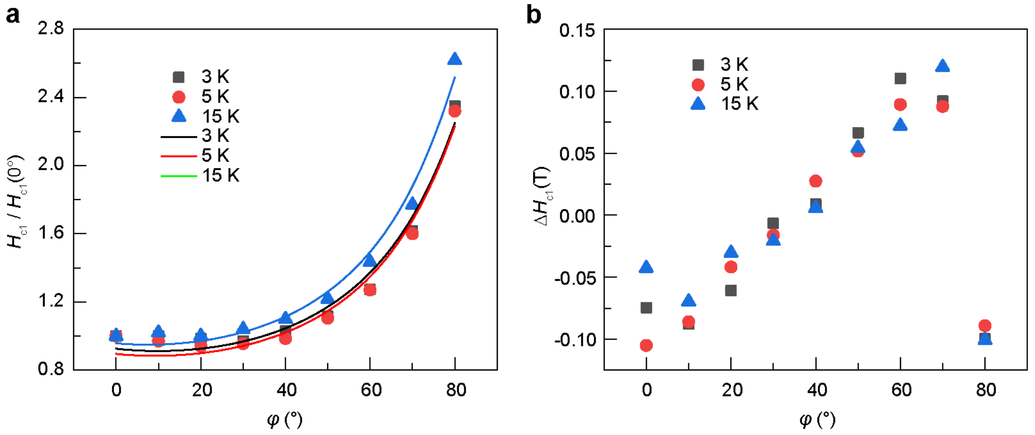

Figure R15. Modified K-model fitting of 2.4 nm CrSb/7 QL $(Bi_{0.19}Sb_{0.81})_2Te_3$ at variable temperatures. **a**, Modified K-model fitting at 3-15 K. **b**, The residuals between the model and the experiment results.

Comment 19: *Present uncertainty propagation from device geometry and contact resistance into Hc1, Hc2, and sigma_xy; confirm that the reported large change in zero-field AHE with composition remains well beyond systematic error bars.*

Response to comment 19:

As the reviewer pointed out, in the six-terminal Hall measurement, the geometry of the device can lead to a R_{xx} component in the transverse signal. As mentioned in the literature[18,19], to eliminate the R_{xx} component caused by the geometric structure, the antisymmetrized process was a standard procedure employed for the Hall signals (R_{xy} = (R_{xy}^{Raw}(+μ_0H)-R_{xy}^{Raw}(-μ_0H))/2). The data presented in our article are all after the antisymmetrization process, meaning that the longitudinal signal caused by the geometric structure had been eliminated. In the Hall measurement, after eliminating the R_{xx} component, the current flows along the longitudinal direction, and the Hall signal is in the transverse direction. No current flows through the voltage terminals, so the contact resistance usually does not produce a voltage drop. Therefore, the R_{xy} is the true anomalous Hall signal. Thus, the H_{c1}, H_{c2}, and zero-field R_{xy} we obtained all come from the true anomalous Hall signal. Accordingly, we emphasized the point in the **Suppl. Section 4**.

Comment 20: *Discuss operational limits and integration: to what extent can the quasi-2D altermagnetic signal survive toward device-relevant temperatures and/or with thicker CrSb; outline a pathway (materials or electrostatic control) to move the demonstrated effects closer to practical operating windows.*

Response to comment 20:

The coercivity produced by this quasi-2D CrSb (e.g., 2.4 nm CrSb/2 QL $(Bi_{0.19}Sb_{0.81})_2Te_3$) can be maintained up to approximately 250K. Thicker CrSb layers, which more closely resemble bulk CrSb, further weaken the quasi-2D characteristics. The exchange coupling can be further enhanced by optimizing interface engineering, and the chances of reaching practical operating windows can be further increased by developing intrinsic alternating magnetic topological insulators. However, behavior at higher than room temperature is an untested territory for future studies. Accordingly, we added it in **the main text (Page 10).**

We thank the reviewer for the comments and trust our revised manuscript is now acceptable for publication.

Response to Reviewer #2 Comments:

General comments:

The authors investigate the anomalous Hall effect (AHE) in quasi-2D CrSb (2.4 nm)/$(Bi_{1-x}Sb_x)_2Te_3$ heterostructures grown by MBE. They demonstrate that growth on the topological insulator induces tensile strain in the CrSb layer due to lattice mismatch, which breaks the collinear altermagnetic spin symmetry, and produces pronounced AHE and tunable spin anisotropy. Magnetization measurements reveal two-step hysteresis loops in thicker TI layers, indicating the coexistence of strain-modified altermagnetic order and interfacial exchange coupling. The AHE strength is shown to be tunable via both the strain and the Fermi-level position of the TI. An effective Hamiltonian model describes how exchange coupling between the altermagnetic order and the TI surface states gives rise to a hybrid AHE.

Overall, I find the paper solid and presenting significant results on quasi-2D interfaces between an altermagnet and a topological insulator.

Response to general comments:

We sincerely thank Reviewer 2 for the strong endorsement of the scientific significance of our manuscript in the quasi-2D interfaces between an altermagnet and a topological insulator. In response to the insightful comments, we have made revisions in the manuscript by providing relevant supporting data to further justify the statements as follows:

1. We performed cross-section STEM (Suppl. Section 2 and Page 4 to Comment 1).

2. We carried out additional TI thickness-dependent, multi-temperature magneto-transport measurements (Suppl. Section 6).

3. We examined reproducibility (Suppl. Section 11 and Page 8).

4. We compared the fitting of the classical K-model, the modified Kondorsky, and the single-domain Stoner–Wohlfarth (Suppl. Section 14 and Page 10).

In addition, we have also made necessary modifications in both the manuscript and the supplementary information in order to further improve the readability and make our work more suitable for the general audience in the magnetic topological insulators research community.

Comment 1: *It highlights the crucial role of strain and exchange coupling in modifying the altermagnetic spin texture, but I think that a more direct experimental verification of the altermagnetic state in strained CrSb would further strengthen the work.*

Response:

We thank the reviewer for the insightful suggestion and carried out STEM to confirm the sample structure of CrSb and TI. As shown in Fig. R16a-b, the results displayed the atomic arrangement of the CrSb and TI layers above and below the interface. Here, we note that ARPES may help provide additional experimental confirmation and perspective. However, in our system, the interface is buried, and the sample surface is covered with a 1-3 nm AlO_x protective capping layer, which may present challenges such as charge accumulation issues and difficulty in detecting the interface for our samples. Nevertheless, the STEM result displayed structural verification of CrSb. Accordingly, we added it to the **Supplementary Section 2** and revised the **main text (Page 4 and Method section)**.

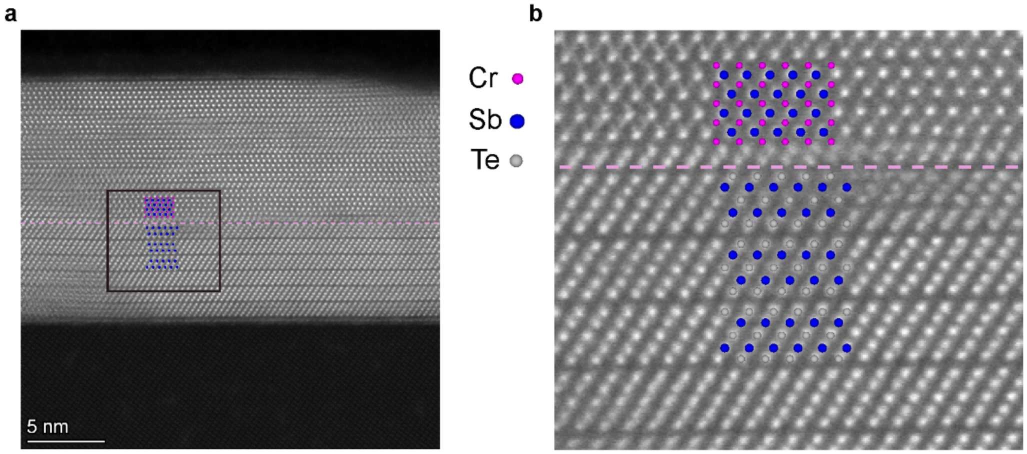

Figure R16. HR-STEM characterization of the CrSb/Sb_2Te_3 thin films. a, Cross-sectional HR-STEM image of CrSb/Sb_2Te_3 heterostructure. **b**, Enlarged the atomic arrangements (captured from the black box of **a**).

References

1. Thole, B., Carra, P., Sette, F. & van der Laan, G. X-ray circular dichroism as a probe of orbital magnetization. *Physical review letters* **68**, 1943 (1992).
2. He, Q. L. *et al.* Topological transitions induced by antiferromagnetism in a thin-film topological insulator. *Phys. Rev. Lett.* **121**, 096802, doi:https://doi.org/10.1103/PhysRevLett.121.096802 (2018).
3. Zhang, H. *et al.* Angle-resolved photoemission spectroscopy. *Nature Reviews Methods Primers* **2**, 54 (2022).
4. Wang, G. *et al.* Atomically smooth ultrathin films of topological insulator Sb2Te3. *Nano Research* **3**, 874-880 (2010).
5. Zhang, T., Ha, J., Levy, N., Kuk, Y. & Stroscio, J. Electric-field tuning of the surface band structure of topological insulator Sb_2Te_3 thin films. *Phys. Rev. Lett.* **111**, 056803 (2013).
6. Nagaosa, N., Sinova, J., Onoda, S., MacDonald, A. H. & Ong, N. P. Anomalous Hall effect. *Rev. Mod. Phys.* **82**, 1539 (2010).
7. Xiao, D., Chang, M.-C. & Niu, Q. Berry phase effects on electronic properties. *Rev. Mod. Phys.* **82**, 1959 (2010).
8. Chang, C. Z. *et al.* Thin films of magnetically doped topological insulator with carrier-independent long-range ferromagnetic order. *Adv. Mater.* **25**, 1065-1070 (2013).
9. Tai, L. *et al.* Distinguishing the two-component anomalous hall effect from the topological Hall effect. *ACS nano* **16**, 17336-17346 (2022).
10. Yu, T. *et al.* Néel vector-dependent anomalous transport in altermagnetic metal CrSb. *npj Quantum Materials* **10**, 47 (2025).
11. Zou, W.-J. *et al.* Enormous Berry-curvature-based anomalous Hall effect in topological insulator $(Bi,Sb)_2Te_3$ on ferrimagnetic europium iron garnet beyond 400 K. *ACS Nano* **16**, 2369-2380, doi:https://doi.org/10.1021/acsnano.1c08663 (2022).
12. Cebollada, F., Rossignol, M., Givord, D., Villas-Boas, V. & González, J. Angular dependence of coercivity in Nd-Fe-B sintered magnets: Proof that coherent rotation is not involved. *Phys. Rev. B* **52**, 13511 (1995).
13. Tan, C. *et al.* Hard magnetic properties in nanoflake van der Waals Fe_3GeTe_2. *Nat. Commun.* **9**, 1554 (2018).
14. Ali, A., Chiang, Y. W. & Santos, R. M. X-ray diffraction techniques for mineral characterization: A review for engineers of the fundamentals, applications, and research directions. *Minerals* **12**, 205 (2022).
15. Regus, M., Kuhn, G., Mankovsky, S., Ebert, H. & Bensch, W. Investigations of the crystallization mechanism of CrSb and $CrSb_2$ multilayered films using in-situ X-ray diffraction and in-situ X-ray reflectometry. *J Solid State Chem.* **196**, 100-109 (2012).
16. Bessas, D. *et al.* Lattice dynamics in Bi_2Te_3 and Sb_2Te_3: Te and Sb density of phonon states. *Phys. Rev. B* **86**, 224301 (2012).
17. Zhong, J., Zhuang, J. & Du, Y. Recent progress on the planar Hall effect in quantum materials. *Chinese Physics B* **32**, 047203 (2023).
18. DeGrave, J. P., Liang, D. & Jin, S. A general method to measure the Hall effect in nanowires: Examples of FeS2 and MnSi. *Nano letters* **13**, 2704-2709 (2013).
19. Rajan, A. Anomalous hall effect and spin-orbit torques in noncollinear antiferromagnetic Mn3Ni0. 35Cu0. 65N thin films. (2025).
20. Rodriguez, J. *et al.* Spin Accumulation based deep MOKE Microscopy. *arXiv preprint arXiv:2511.13468* (2025).

Tailoring topological altermagnetic spin texture via interfacial exchange coupling in quasi-2D CrSb/(Bi, Sb)$_2$Te$_3$ thin film

Peng Chen[1], Josep Ingla-Aynés[1], Chen Chang[2], Eugene Park[2], Don Heiman[1,3], Frances M. Ross[2], Hang Chi[4,5,6], & Jagadeesh S. Moodera[1,7]

[1]*Francis Bitter Magnet Laboratory, Plasma Science and Fusion Center, Massachusetts Institute of Technology, Cambridge, Massachusetts 02139, USA*

[2]*Department of Materials Science and Engineering, Massachusetts Institute of Technology, Cambridge, Massachusetts 02139, USAs*

[3]*Department of Physics, Northeastern University, Boston, Massachusetts 02115, USA*

[4]*Department of Physics, University of Ottawa, Ottawa, Ontario K1N 6N5, Canada*

[5]*School of Electrical Engineering and Computer Science, University of Ottawa, Ottawa, Ontario K1N 6N5, Canada*

[6]*Nexus for Quantum Technologies, University of Ottawa, Ottawa, Ontario K1N 6N5, Canada*

[7]*Department of Physics, Massachusetts Institute of Technology, Cambridge, Massachusetts 02139, USA*

Response to Reviewer 1 Comments:

General comments:

The author addressed the questions very well and I have no concern now and would like to recommend this paper to be publish in Nature Communications!

Response: We thank Reviewer 1 for recommending this paper to be published.

Response to Reviewer 2 Comments:

General comments:

I think the authors have addressed all reasonable experimental concerns raised by the reviewers, providing additional data and clarifications within the practical limitations of their system. While some direct measurements are not feasible due to sample constraints, the combination of transport data, structural characterization, and comparison with previous literature provides a coherent and convincing picture. Overall, the manuscript presents novel and significant results, and I consider it suitable for publication.

Response: We thank Reviewer 2 for supporting our manuscript for publication.

Response to Reviewer 3 Comments:

General comments:

This manuscript systematically investigates MBE-grown quasi-2D CrSb (2.4 nm)/(Bi1-xSbx)2Te3 heterostructures, proposing that interfacial tensile strain breaks the collinear altermagnetic symmetry of CrSb to induce a pronounced anomalous Hall effect (AHE). By fine-tuning the topological insulator (TI) thickness and Sb doping ratio, the authors observe a remarkable transition from a single- to a two-step AHE hysteresis loop. Supported by an effective Hamiltonian and a modified Kondorsky model, this dual-component behavior is convincingly attributed to the coexistence of strain-broken intrinsic altermagnetism and interfacial topological surface state exchange coupling. Although the work is outstanding in its experimental design and comprehensiveness of data, I find that several critical issues remain regarding the "closing of the loop" between macroscopic transport phenomena and the microscopic magnetic structure/theoretical models. Therefore, I recommend major revisions to fully address the following critical issues.

Response: We thank Reviewer 3 for recognizing our work as “outstanding” with “remarkable” experimental observation and “convincing” attribution in its mechanism. Following the reviewer’s insightful suggestions, we have made revisions in the manuscript and provided relevant data to clarify the statements further as follows:

1. We provided a qualitative, mechanism-focused physical discussion on the link between interfacial strain and the emergence of net magnetic moment (Pages 4-5 to Comment 1).

2. We briefly discussed the influence of an in-plane exchange field component on the Hamiltonian (Page 7 and Suppl. Section 9 to Comment 2).

3. We added the angle-dependent anomalous Hall signal from 20-150 K and summarized the fitted tilt angle (Page 10 and Suppl. Section 14 to Comment 3).

4. We further carried out the exchange bias measurements (Comment 4).

5. We performed magnetic-field-dependent longitudinal signal at different temperatures (Page 6 and Suppl. Section 6 to Comment 5).

Additionally, we have also made necessary modifications in the manuscript to further enhance the readability and ensure that our work is more accessible to the general audience in the condensed matter physics community.

Comment 1: *Considering that conducting precise first-principles calculations on alloy superlattices is rather complex, could the authors provide a qualitative, mechanism-focused physical discussion (based on crystal field theory or a simplified spin-orbit coupling Hamiltonian) on the causal link between interfacial strain and the emergence of net magnetic moment? Specifically, how does in-plane tensile strain alter specific Cr-Sb bond lengths or angles, thereby preferentially breaking the initially compensated g-wave altermagnetic symmetry rather than causing other forms of magnetic disorder?*

Response to comment 1:

In an ideal altermagnet (e.g., bulk case), a pair of magnetic sublattices (A and B) mutually compensate in real space (e.g., the moment of magnetic sublattices $m_A = -m_B$), where the two magnetic sublattices share the same lattice environment. However, in ultrathin films, especially heterostructures, lattice constant mismatch introduces interfacial strain. In CrSb/TI, the in-plane lattice constant of CrSb is smaller than that of TI, causing tensile stress on the interfacial CrSb, resulting in in-plane lattice stretching and out-of-plane lattice compression. This leads to an increase in the in-plane Cr-Sb bond length and a decrease in the out-of-plane, causing a change in the crystal field and modulation of the hopping amplitude, making $m_A \neq -m_B$, thereby producing the net magnetic moment. The strain introduced by epitaxial growth typically exhibits coherent distortion, systematically modifying exchange interactions[1-3]. Therefore, in-plane tensile strain that modifies the Cr-Sb bond geometry can break the magnetic compensation between two sublattices. Accordingly, we added some of this discussion in the main text (**Pages 4-5**).

Comment 2: *The effective Hamiltonian model in the main text only considers the out-of-plane component of the exchange field from CrSb. Given that the modified Kondorsky model reveals a tilted effective moment, could the authors briefly discuss the influence of an in-plane exchange field component on the Hamiltonian eigenstates and the resulting AHE, to ensure consistency between the two theoretical analyses?*

Response to comment 2:

When considering an in-plane magnetization to the surface states of a TI, it causes the Dirac cone of the TI to undergo a shift or tilt in k-space[4], rather than opening a magnetic gap that generates AHE. In the associated Hamiltonian model, no σ_z term is generated. In the tilted spin texture, the more significance is the generation of net out-of-plane magnetization, which causes the TI surface states to lift the Kramers degeneracy at any Dirac points and further generate σ_z terms[4]. Accordingly, we have incorporated a discussion of the in-plane field into our discussion about the Hamiltonian model (**Page 7** and **Suppl. Section 9**).

Comment 3: *The authors introduced the equivalent tilt angle in the modified Kondorsky model to perfectly fit the angle-dependent Hc1 data, showing fitting results between 3 K and 15 K in their rebuttal. Given that*

the equivalent tilt angle is considered a comprehensive manifestation of strain and interfacial coupling, could the authors systematically analyze and discuss the specific evolutionary trend of this fitted the equivalent tilt angle parameter as the temperature increases? Does this trend align with the thermal attenuation of the interfacial coupling strength as it approaches 20 K (the critical temperature where the two-step behavior disappears)?

Response to comment 3:

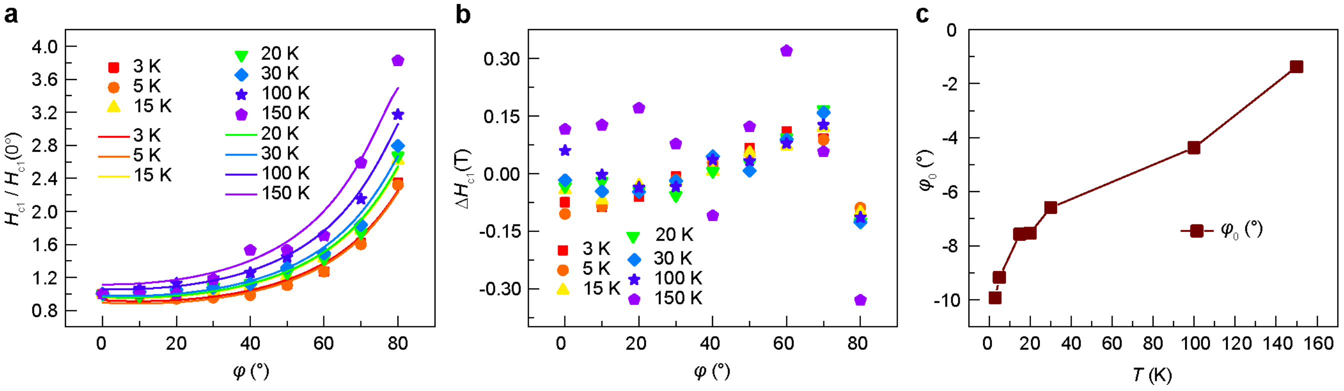

Figure R1. Modified K-model fitting of 2.4 nm CrSb/7 QL $(Bi_{0.19}Sb_{0.81})_2Te_3$ at variable temperatures. **a**, Modified K-model fitting at 3-150 K. **b**, The difference between the model and the experiment results. c, temperature-dependent fitted tile angles φ_0.

Based on the third reviewer's suggestion, we measured angle-dependent Hall signals from 20 K to 150 K and performed the fitting using the modified K-model (Fig. R1a). Figure R1b shows the residuals between the fitting and the experiment, where most residual values are small within ±0.12 T. The trend of the fitted tilt angle φ_0 with temperature is summarized in Fig. R1c. The overall trend is that φ_0 gradually decreases and approaches 0 with increasing temperature. Two different values of slope are observed below and above ~20 K, which seems to suggest different rates of decrease in the tilt angle, in corroboration with the transition from two-step to single AHE around 20 K. Accordingly, we revised the main text (**Page 10**) and added the fitting and the trend of tilt angle to **Suppl. Section 14.**

Comment 4: *Since the system contains a strain-broken altermagnetic dominant phase (H_{c1}) and a weaker interfacial topological surface phase (H_{c2}), this constitutes a typical magnetically coupled interface. In the current minor-loop cycling tests, the reversals of these two phases appear quite independent. Could the authors discuss whether any exchange bias effects (i.e., a horizontal shift of the magnetic hysteresis loop*

along the magnetic field axis) caused by the interaction between these two phases were observed under field-cooling conditions or specific bias magnetic fields?

Response to comment 4:

To address this insightful question, we cooled down the device to 1.7 K in magnetic fields at 0, 3, and -3 T, respectively, and tested the associated anomalous Hall signals. As shown in Fig. R2, the anomalous Hall signal didn't show a significant shift along the magnetic field direction in both the zero magnetic field and the case of cooling with the magnetic fields. This result rules out the existence of exchange bias.

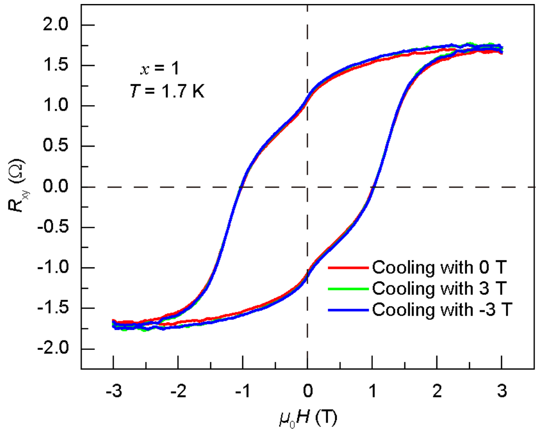

Figure R2. The AHE resistance of 2.4 nm CrSb/7 QL Sb_2Te_3 cooled down with 0, 3, and -3 T.

Comment 5: *Currently, the main evidence for interfacial topological exchange coupling is centered on the low-coercivity branch (H_{c2}) in the transverse anomalous Hall resistance (R_{xy}). To further corroborate this coupling effect from a complementary perspective, could the authors examine and present whether there are any subtle anomalous features in the temperature or magnetic-field-dependent curves of the longitudinal resistance (R_{xx}) near the transition temperature (~20 K) that correspond to the decoupling of the surface states or magnetic scattering?*

Response to comment 5:

We obtained the longitudinal signal under varying temperatures, as shown in Fig. R3. The anomalous features are evident at 1.7 K, gradually decreasing with increasing temperature and disappearing at ~10-15 K. Although this temperature deviates slightly from the temperature at which H_{c2} vanishes in R_{xy} (~20 K), a transition from a hybrid R_{xx} to a single R_{xx} is observed. As the temperature rises further, the butterfly-type magnetic signal in R_{xx} disappears at ~ 250 K, which is consistent with the temperature at which H_{c1} vanishes. Accordingly, we added R_{xx} results to the **Suppl. Section 6** and revised the main text (**Page 6**).

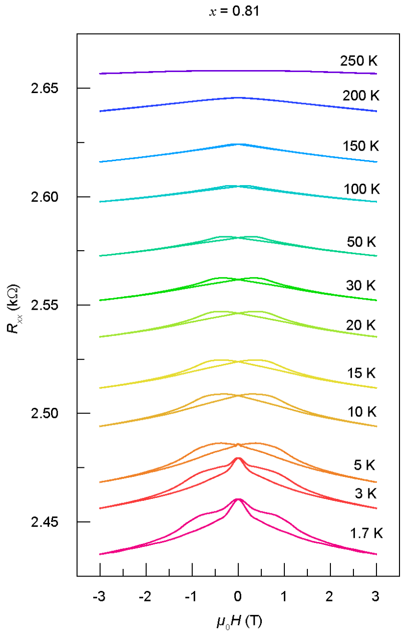

Figure R3. The longitudinal signals of 2.4 nm CrSb/7 QL $(Bi_{0.19}Sb_{0.81})_2Te_3$ at different temperatures.

References:

1 Jeong, S. G. *et al.* Metallicity and anomalous Hall effect in epitaxially strained, atomically thin RuO2 films. *Proceedings of the National Academy of Sciences* **122**, e2500831122 (2025).

2 Lupascu, A. *et al.* Tuning magnetic coupling in Sr 2 IrO 4 thin films with epitaxial strain. *Physical Review Letters* **112**, 147201 (2014).

3 Disa, A. S. *et al.* Polarizing an antiferromagnet by optical engineering of the crystal field. *Nature Physics* **16**, 937-941 (2020).

4 Zhang, F., Kane, C. L. & Mele, E. J. Surface state magnetization and chiral edge states on topological insulators. *Phys. Rev. Lett.* **110**, 046404 (2013).

Tailoring topological altermagnetic spin texture via interfacial exchange coupling in quasi-2D $CrSb/(Bi, Sb)_2Te_3$ thin film

Peng Chen[1], Josep Ingla-Aynés[1], Chen Chang[2], Eugene Park[2], Don Heiman[1,3], Frances M. Ross[2], Hang Chi[4,5,6], & Jagadeesh S. Moodera[1,7]

[1]*Francis Bitter Magnet Laboratory, Plasma Science and Fusion Center, Massachusetts Institute of Technology, Cambridge, Massachusetts 02139, USA*

[2]*Department of Materials Science and Engineering, Massachusetts Institute of Technology, Cambridge, Massachusetts 02139, USAs*

[3]*Department of Physics, Northeastern University, Boston, Massachusetts 02115, USA*

[4]*Department of Physics, University of Ottawa, Ottawa, Ontario K1N 6N5, Canada*

[5]*School of Electrical Engineering and Computer Science, University of Ottawa, Ottawa, Ontario K1N 6N5, Canada*

[6]*Nexus for Quantum Technologies, University of Ottawa, Ottawa, Ontario K1N 6N5, Canada*

[7]*Department of Physics, Massachusetts Institute of Technology, Cambridge, Massachusetts 02139, USA*

Response to Reviewer 3 Comment:

General comments:

The authors have thoroughly addressed all the raised concerns, and the manuscript is now suitable for publication.

Response: We thank Reviewer 3 for recommending this paper to be published.
